# Supplementary material for: Companion restrictions in the emergency department during COVID-19: physician perceptions from the Western Cape, South Africa
Source: BMJ Open. 2023 May 5;13(5):e070982. doi: 10.1136/bmjopen-2022-070982 (PMC10163331; doi:10.1136/bmjopen-2022-070982)
Supplement: Supplementary data [file bmjopen-2022-070982supp001.pdf]

APPENDIX 1: Interview Guide

Thank you for accepting to take part in this interview. Our goal is to learn about your perspective of the changing processes in your trauma care facility during COVID-19, with a particular focus on any changes that have occurred due to no hospital visitors. The interviews will be conducted by Lauren Wiebe, a Master's student from Canada in Public Health Sciences at Karolinska Institutet under the supervision of Dr Helle Alvesson and Dr Willem Stassen.

Experience

A. Prior to COVID-19, what was your role in this unit, and can you describe a bit about your work? For example, what kind of tasks did you do? Who in the unit did you work with? What kind of patients did you see?
B. Before COVID-19, approximately how many patients did you see in a day? How many do you see now? Approximately what month do you feel this changed?
C. Besides COVID-19, during your time working at this hospital have you ever had to work through a secondary emergency before e.g. epidemic, natural disasters, conflict? Have you had this type of experience at another health facility?

Changing practices during COVID-19

A. Can you list some of the practices that have changed in your unit since COVID-19?

→ *The answers from this list can be used as prompts for the below questions. Examples: intake/triage process, referral system, caregiver presence, health seeking behaviour, teamwork among the unit, resource distribution*

B. Please describe what aspects of these changes you value? What aspects do you dislike?
C. Please describe how you learned to adapt to the changing practices? For example, access to training, protocol documents or team meetings.

Making sense of the changes

A. How do these changes compare to your practices before? Can you please provide me with an example?
B. Do you feel like the changes that have occurred are important? If yes, please describe.
C. Do you think your colleagues share an understanding of these changes and their importance? If yes, please describe what influenced this shared understanding? If no, please describe why there are perceived differences?

Participating in the changes

A. In your opinion, do the staff in your unit all equally participate in these changes? If no, what do you think are the main reasons people are not participating in these changes?
B. Are there individuals in the unit who are promoting and supporting the team in these changes? For example, a management team, chief doctor etc.
    a. If yes, please describe the ways they promote/support the unit during COVID?
    b. If yes, did they hold these leadership positions prior to COVID-19?
    c. If there is no central promotor/enforcer, what do you think motivates the staff to participate?
C. Do you feel the new practices are sustainable moving forward? If yes, please explain.

Operationalization of the changes

A. Do you think all staff members in your unit are equally affected by the changes from COVID-19?
B. Since COVID-19, have you seen a change in how the resources are distributed?
C. How have relationships and interactions in your job changed with the changing practices?

Monitoring and assessment of the changes

A. In your opinion have these new practices been effective? Has anything improved from how they were practiced pre-covid? If so, can you give me an example?
B. Please describe how these changes have affected how the medical team works together?
C. How do you feel these changes have affected you individually and your job tasks?
D. Among these changes, what practices do you think could be improved? If so, can you give me an example?

**If not previously discussed-* We would like to ask you a few more questions specifically about the changes you experienced with no caregivers being able to accompany the patient into the health facility.

A. In general, did you notice any changes to care with no caregivers/family being able to accompany the patient?
B. How did the restrictions on family members affect your communication with the patient?
    a. How did you communicate the health news to the families?
    b. How did it influence how you communicated with your colleagues?
C. How did you feel about no caregivers/family members being allowed?
D. What do you think the patients felt having no caregiver with them?
E. In your opinion, what do you feel could have made this better?

Final Questions

A. Are there any other comments you would like to share related to your personal experience working with COVID-19 that have not been addressed in this interview?
B. Do you have any final questions for me?

Questionnaire to be completed before or after the interview:

A. What is your age?
B. What is your identified gender?
C. What is your profession and specialization?
D. How many years have you been working in the health profession?
E. How many years have you been working at this hospital?
F. During the COVID-19 outbreak, were you working part-time or full-time?
