# Supplementary material for: Companion restrictions in the emergency department during COVID-19: physician perceptions from the Western Cape, South Africa
Source: BMJ Open. 2023 May 5;13(5):e070982. doi: 10.1136/bmjopen-2022-070982 (PMC10163331; doi:10.1136/bmjopen-2022-070982)
Supplement: Supplementary data [file bmjopen-2022-070982supp004.pdf]

APPENDIX 4: Consent Letter Study II

PART 1: INFORMED CONSENT FORM

The perceptions and experiences of emergency medical staff on the impact of COVID-19 on trauma emergency care service provision in the Western Cape province of South Africa

Investigators and affiliations
Dr Willem Stassen, Div. Emergency Medicine, University of Cape Town
Prof Helle M. Alvesson, Dept. Global Public Health, Karolinska Institutet
Ms Elzarie Theron, Div. Emergency Medicine, University of Cape Town
Ms Lauren Wiebe, Dept. Global Public Health, Karolinska Institutet

You are invited to participate in a study conducted by the Division of Emergency Medicine at the University of Cape Town and the Department of Global Public Health at the Karolinska Institutet, Sweden.

COVID-19 was declared a global pandemic in March 2020 following a cluster of pneumonia cases in Wuhan, China. Caused by severe acute respiratory syndrome Coronavirus -2 (SARS-CoV-2), COVID-19 continues to spread across the globe. The purpose of this study is to understand the perceptions of emergency care personnel in the Western Cape on the changes in care provision due to COVID-19. We aim to learn from your perspective including, what trauma care processes and practices changed due to COVID-19 and the various ways you had to adjust, specifically inquiring about your perceptions regarding no hospital visitors and escorts. Understanding this perspective can mitigate operational consequences of the COVID-19 pandemic and in the planning for future pandemics. You were selected to participate in this study because you are working clinically on the frontline during the COVID-19 pandemic. Doctors and nurses regardless of baseline qualification are eligible. We aim to recruit a maximum of 20 frontline clinical providers in this study.

Voluntary Participation
Whether you decide to participate in the study or not is entirely your choice and voluntary. There will be no consequences to you if you decide not to participate. You may also decide to change your mind and may withdraw from participating, even if you agreed to it at an earlier stage.

Description of the Process
The interviews will take place over a period of 1-3 months in February-April 2021 and we will require your participation at one session within this period. If you choose to participate in this study, your interview session will take approximately 30-60 minutes. We will require the following:

- A short open-ended survey will be administered and used to collect biographical data at the onset of the study.
- A one-on-one online interview and will take approximately 30 to 60 minutes

Risks or Benefits

Direct benefits to you as an individual could include an opportunity to share your experiences and emotions during the COVID-19 pandemic. It is envisioned that the findings of this study could contribute towards an emergency care system to the advantage of the emergency care community.

Participation in the study could elicit negative emotions due to the sensitive nature of the topic and the emotional experiences you may have with it. In the event that you are experiencing discomfort as a result of any of the research processes, we will support you to obtain the psychological counselling / assistance you require. If you experience psychological discomfort during an interview, please bring this to the interviewer's attention and s/he will support you to obtain the assistance you require.

Confidentiality

All entries will be anonymised during the transcription, analysis and reporting processes. We will use a pseudonym to identify you. The researchers undertake to keep any information provided herein confidential, not to let it out of our possession and to report on the findings from the perspective of the participating group and not from the perspective of an individual.

Right to Refuse or Withdraw

You are not obliged to participate in this study if you do not want to do so. You may withdraw your participation at any time if you choose to do so, up until reporting of analyses. It is entirely your choice and no negative consequences will be incurred if you should choose not to participate or withdraw.

Future research

Anonymised transcribed data will be stored and will later be analysed to inform the initial phase of theory generation, thus contributing towards further related research. Such a theory can assist us in the future to understand the perceptions and experiences of frontline workers and to create systems that may better support them during pandemics.

Who to Contact

The research was reviewed and approved by the Human Research Ethics Committee of the University of Cape Town. The researcher team can be contacted during office hours at elzarie.theron@uct.ac.za. The UCT's Faculty of Health Sciences Human Research Ethics Committee can be contacted on 021 406 6338 in case you have any ethical concerns or questions about your rights or welfare as a participant on this research study.

Wiebe LE, *et al. BMJ Open* 2024; 13:e070982. doi: 10.1136/bmjopen-2022-070982

PART 2: CONSENT

The perceptions and experiences of emergency medical staff on the impact of COVID-19 on trauma emergency care service provision in the Western Cape province of South Africa

I have read the foregoing information, or it has been read to me. I have had the opportunity to ask questions about it and any questions that I have had has been answered to my satisfaction. I consent voluntarily to participate as a participant in this interview. I understand that I may be identifiable.

The purpose of the recordings and the details of its storage and future analysis has been described to me. I have been offered to have any questions that I may have on the recording of the interview – answered and explained. I am giving consent voluntarily and I have been given a copy of this consent form.

Print Name of Participant: ____

Signature of Participant: ____

Date: ____

A copy of this informed consent form has been provided to the participant.

Print Name of Researcher: _______________________________________

Signature of Researcher: ____

Date: ____

Wiebe LE, *et al. BMJ Open* 2024; 13:e070982. doi: 10.1136/bmjopen-2022-070982
