# Supplementary material for: Companion restrictions in the emergency department during COVID-19: physician perceptions from the Western Cape, South Africa
Source: BMJ Open. 2023 May 5;13(5):e070982. doi: 10.1136/bmjopen-2022-070982 (PMC10163331; doi:10.1136/bmjopen-2022-070982)
Supplement: Supplementary data [file bmjopen-2022-070982supp005.pdf]

APPENDIX 5: Consent Letter Study I

Lived experiences of emergency care personnel in the Western Cape, South Africa during the COVID-19 pandemic: a longitudinal hermeneutic phenomenological study

Investigators and affiliations

1. **Willem Stassen**, Division of Emergency Medicine, University of Cape Town
2. **Elzarie Theron**, Division of Emergency Medicine, University of Cape Town
3. **Helena Erasmus**, Department of Psychology, University of South Africa
4. **Craig Wylie**, Division of Emergency Medicine, Stellenbosch University
5. **Waseela Khan**, Division of Emergency Medicine, University of Cape Town
6. **Heike Geduld**, Division of Emergency Medicine, Stellenbosch University

You are invited to participate in a study conducted by the Division of Emergency Medicine at the University of Cape Town.

COVID-19 was declared a global pandemic in March 2020 following a cluster of pneumonia cases in Wuhan, China. Caused by severe acute respiratory syndrome Coronavirus -2 (SARS-CoV-2), COVID-19 continues to spread across the globe. The purpose of this study is to understand the lived experiences of emergency care personnel in the Western Cape. Understanding this perspective is necessary to mitigate adverse psychological and operational consequences of the COVID-19 pandemic by informing strategies to maintain appropriate emergency medical care through the life cycle of this pandemic, and in the planning for future pandemics. You were selected to participate in this study because you are working clinically on the frontline during the COVID-19 pandemic. Doctors, nurses and prehospital staff regardless of baseline qualification are eligible. We aim to recruit 12-16 frontline clinical providers in this study.

Voluntary Participation

Whether you decide to participate in the study or not is entirely your choice and voluntary. There will be no consequences to you if you decide not to participate. You may also decide to change your mind and may withdraw from participating, even if you agreed to it at an earlier stage.

Description of the Process

The study will take place over a period of three months and we will require your participation at various intervals during this period. If you choose to participate in this study, it will take up no more than 60 minutes of your time each week for the duration of the study. We will require the following:

- A short interviewer-administered open-ended survey will be used to collect biographical data at the onset of the study. The survey will be approximately 15 minutes long and will be conducted online, using an online communication tool for video calls.
- You will be asked to share their work schedule during the first interview and follow-up interviews and deadlines for data collection points will be planned accordingly.
- You will be asked to record a 15-30 minute voice recording reflecting on you lived experiences to date in the context of COVID-19. This should be submitted prior to their first pre-shift recording.

- You will be asked to record three to five-minute voice recordings on the following recommended times: 1) prior to a shift, 2) directly after a shift, and 3) 24 to 48 hours after the last shift in a cycle.
- One-on-one interviews will be conducted twice a month for the duration of the study. An online communications platform will be used to conduct the interviews, which will be approximately 40 to 60 minutes in length.
- You will be asked to complete the WHO (Five) Well-being scale every second week. The questionnaire will be available online and consists of a six-point Likert scale measuring positive and negative mental wellbeing.

Risks or Benefits

Direct benefits to you as an individual could include an opportunity to share your experiences and emotions during the COVID-19 pandemic. It is envisioned that the findings of this study will could contribute towards an emergency care system to the advantage of the emergency care community.

You will be reimbursed for the internet data that you use for the study, should you need it.

It is anticipated that the information we gain from this study will help us to reduce adverse psychological and operational consequences of the COVID-19 pandemic by informing strategies to maintain appropriate emergency medical care through the life cycle of this pandemic, and in the planning of future pandemics.

Participation in the study could elicit negative emotions or psychological responses. To monitor this and provide you with the assistance you need in such an event, you will be asked to end your reflections with an indication of how you are currently coping on a scale from 1 to 5 and your need for psychological assistance. In the event that you are experiencing discomfort as a result of any of the research processes, we will support you to obtain the psychological counselling / assistance you require. If you experience psychological discomfort during an interview, please bring this to the interviewer’s attention and s/he will support you to obtain the assistance you require.

Confidentiality

In order to ensure that we can link subsequent entries and interviews to previously submitted entries, we need to link your identity. However, all entries will be anonymised during the transcription, analysis and reporting processes. We will use a pseudonym to identify you.

The researchers undertake to keep any information provided herein confidential, not to let it out of our possession and to report on the findings from the perspective of the participating group and not from the perspective of an individual.

Right to Refuse or Withdraw

You are not obliged to participate in this study if you do not want to do so. You may withdraw your participation at any time if you choose to do so, up until reporting of analyses. It is entirely your choice, and no negative consequences will be incurred if you should choose not to participate or withdraw.

Future research

Anonymised transcribed data will be stored and will later be analysed to inform the initial phase of theory generation, thus contributing towards further related research. Such a theory can assist us in the future to understand the experiences of frontline workers and to create systems that may better support them during pandemics.

Who to Contact
The research was reviewed and approved by the Human Research Ethics Committee of the University of Cape Town. The researcher team can be contacted during office hours at elzarie.theron@uct.ac.za. Should you have any questions regarding the ethical aspects of the study, you can contact the Human Research Ethics Committee of the University of Cape Town, South Africa; contact Tel: +27 21 650 3002, Email: hrec-enquiries@uct.ac.za.

Part 2: Consent

Lived experiences of emergency care personnel in the Western Cape, South Africa during the COVID-19 pandemic: a longitudinal hermeneutic phenomenological study

I have read the foregoing information, or it has been read to me. I have had the opportunity to ask questions about it and any questions that I have had has been answered to my satisfaction. I consent voluntarily to participate as a participant in this interview. I understand that I may be identifiable.

The purpose of the recordings and the details of its storage and future analysis has been described to me. I have been offered to have any questions that I may have on the recording of the interview – answered and explained. I am giving consent voluntarily and I have been given a copy of this consent form.

Print Name of Participant: ____
Signature of Participant: ____
Date: ____

A copy of this informed consent form has been provided to the participant.

Print Name of Researcher: _______________________________________
Signature of Researcher: ____
Date: ____
