# Supplementary material for: Companion restrictions in the emergency department during COVID-19: physician perceptions from the Western Cape, South Africa
Source: BMJ Open. 2023 May 5;13(5):e070982. doi: 10.1136/bmjopen-2022-070982 (PMC10163331; doi:10.1136/bmjopen-2022-070982)
Supplement: Supplementary data [file bmjopen-2022-070982supp002.pdf]

APPENDIX 2: Ethical Approval Study II

UNIVERSITY OF CAPE TOWN
Faculty of Health Sciences
Human Research Ethics Committee

Room G50- Old Main Building
Groote Schuur Hospital
Observatory 7925
Telephone [021] 406 6492
Email: hrec-enquiries@uct.ac.za
Website: www.health.uct.ac.za/fhs/research/humanethics/forms

23 February 2021

HREC REF: 052/2021

Dr W Stassen
Division of Emergency Medicine
F51, OMB
Email: willem.stassen@uct.ac.za

Dear Dr Stassen

PROJECT TITLE: THE PERCEPTIONS AND EXPERIENCES OF EMERGENCY MEDICAL STAFF ON THE IMPACT OF COVID-19 ON TRAUMA EMERGENCY CARE SERVICE PROVISION IN THE WESTERN CAPE PROVINCE OF SOUTH AFRICA

Thank you for your response letter, addressing the issues raised by the Faculty of Health Sciences Human Research Ethics Committee (HREC).

It is a pleasure to inform you that the HREC has **formally approved** the above-mentioned study, subject to the following: -

1. There is one outstanding issue. The section on Reflexivity (which seems to be in the wrong place, there is a section on the one visiting master's student. (See below)
   Reflexivity
   Lauren Wiebe has a bachelor's degree in Public Health and Political Science from Vancouver, Canada and is currently a Master's in Public Health Sciences student at Karolinska Institutet. The majority of her experience surrounds public health policy for mental health and infectious diseases for populations in Canada. She has no direct experience working in or with trauma healthcare, reducing preconceptions. However, preconceptions around the role of health workers during the pandemic are that changes have occurred within the trauma unit, potentially in the areas such as teamwork, patient care, protocols and resource distribution.
   Can the researchers please tidy this up and put reflexivity together with the section on credibility and trustworthiness; and put the detail about Lauren in the appropriate place.

This approval is subject to strict adherence to the HREC recommendations regarding research involving human participants during COVID -19, dated 17 March 2020 & 06 July 2020.

Approval is granted for one year until the 28 February 2022.

Please submit a progress form, using the standardised Annual Report Form if the study continues beyond the approval period. Please submit a Standard Closure form if the study is completed within the approval period.
(Forms can be found on our website: www.health.uct.ac.za/fhs/research/humanethics/forms)

Please quote the HREC REF 052/2021 in all your correspondence.

Please note that the ongoing ethical conduct of the study remains the responsibility of the principal investigator.

HREC/REF 052/2021sa
