# Supplementary material for: Companion restrictions in the emergency department during COVID-19: physician perceptions from the Western Cape, South Africa
Source: BMJ Open. 2023 May 5;13(5):e070982. doi: 10.1136/bmjopen-2022-070982 (PMC10163331; doi:10.1136/bmjopen-2022-070982)
Supplement: Supplementary data [file bmjopen-2022-070982supp003.pdf]

APPENDIX 3: Ethical Approval Dataset I

UNIVERSITY OF CAPE TOWN
Faculty of Health Sciences
Human Research Ethics Committee

Room G50- Old Main Building
Groote Schuur Hospital
Observatory 7925
Telephone [021] 406 6492
Email: hrec-enquiries@uct.ac.za
Website: www.health.uct.ac.za/fhs/research/humanethics/forms

06 August 2020

HREC REF: 386/2020

Dr W Stassen
Division of Emergency Medicine
c/o Ms Vathiswa Mzamo
F-51, OMB
Email: willem.stassen@uct.ac.za

Dear Dr Stassen

PROJECT TITLE: LIVED EXPERIENCES OF EMERGENCY CARE PERSONNEL IN THE WESTERN CAPE, SOUTH AFRICA DURING THE COVID-19 PANDEMIC: A LONGITUDINAL HERMENEUTIC PHENOMENOLOGICAL STUDY

Thank you for submitting your study to the Faculty of Health Sciences Human Research Ethics Committee (HREC) for review.

It is a pleasure to inform you that the HREC has **formally approved** the above-mentioned study.

This approval is subject to strict adherence to the HREC recommendations regarding research involving human participants during COVID -19, dated 17 March 2020 & 06 July 2020.

Approval is granted for one year until the 30 August 2021.

Please submit a progress form, using the standardised Annual Report Form if the study continues beyond the approval period. Please submit a Standard Closure form if the study is completed within the approval period.
(Forms can be found on our website: www.health.uct.ac.za/fhs/research/humanethics/forms)

Please quote the HREC REF in all your correspondence.

Please note that the ongoing ethical conduct of the study remains the responsibility of the principal investigator.

Please note that for all studies approved by the HREC, the principal investigator **must** obtain appropriate institutional approval, where necessary, before the research may occur.

Yours sincerely

PROFESSOR M BLOCKMAN
CHAIRPERSON, FHS HUMAN RESEARCH ETHICS COMMITTEE

HREC 386/2020sa
