# Supplementary material for: Companion restrictions in the emergency department during COVID-19: physician perceptions from the Western Cape, South Africa
Source: BMJ Open. 2023 May 5;13(5):e070982. doi: 10.1136/bmjopen-2022-070982 (PMC10163331; doi:10.1136/bmjopen-2022-070982)
Supplement: Author's manuscript [file bmjopen-2022-070982.draft_revisions.pdf]

BMJ Open is committed to open peer review. As part of this commitment we make the peer review history of every article we publish publicly available.

When an article is published we post the peer reviewers' comments and the authors' responses online. We also post the versions of the paper that were used during peer review. These are the versions that the peer review comments apply to.

The versions of the paper that follow are the versions that were submitted during the peer review process. They are not the versions of record or the final published versions. They should not be cited or distributed as the published version of this manuscript.

BMJ Open is an open access journal and the full, final, typeset and author-corrected version of record of the manuscript is available on our site with no access controls, subscription charges or pay-per-view fees (http://bmjopen.bmj.com).

If you have any questions on BMJ Open's open peer review process please email info.bmjopen@bmj.com

BMJ Open

Companion Restrictions in the Emergency Department during COVID-19: Physician Perceptions from the Western Cape, South Africa

Journal:	*BMJ Open*
Manuscript ID	bmjopen-2022-070982
Article Type:	Original research
Date Submitted by the Author:	26-Dec-2022
Complete List of Authors:	Wiebe, Lauren; Karolinska Institutet, Department of Global Public Health<br>Alvesson, Helle; Karolinska Institutet, Department of Global Public Health<br>Stassen, Willem; University of Cape Town, Division of Emergency Medicine
Keywords:	COVID-19, ACCIDENT & EMERGENCY MEDICINE, Protocols & guidelines < HEALTH SERVICES ADMINISTRATION & MANAGEMENT, QUALITATIVE RESEARCH

SCHOLARONE™
Manuscripts

I, the Submitting Author has the right to grant and does grant on behalf of all authors of the Work (as defined in the below author licence), an exclusive licence and/or a non-exclusive licence for contributions from authors who are: i) UK Crown employees; ii) where BMJ has agreed a CC-BY licence shall apply, and/or iii) in accordance with the terms applicable for US Federal Government officers or employees acting as part of their official duties; on a worldwide, perpetual, irrevocable, royalty-free basis to BMJ Publishing Group Ltd ("BMJ") its licensees and where the relevant Journal is co-owned by BMJ to the co-owners of the Journal, to publish the Work in this journal and any other BMJ products and to exploit all rights, as set out in our licence.

The Submitting Author accepts and understands that any supply made under these terms is made by BMJ to the Submitting Author unless you are acting as an employee on behalf of your employer or a postgraduate student of an affiliated institution which is paying any applicable article publishing charge ("APC") for Open Access articles. Where the Submitting Author wishes to make the Work available on an Open Access basis (and intends to pay the relevant APC), the terms of reuse of such Open Access shall be governed by a Creative Commons licence – details of these licences and which Creative Commons licence will apply to this Work are set out in our licence referred to above.

Other than as permitted in any relevant BMJ Author's Self Archiving Policies, I confirm this Work has not been accepted for publication elsewhere, is not being considered for publication elsewhere and does not duplicate material already published. I confirm all authors consent to publication of this Work and authorise the granting of this licence.

For peer review only

Companion Restrictions in the Emergency Department during COVID-19: Physician Perceptions from the Western Cape, South Africa

For peer review only

Lauren E Wiebe[1], Helle Mölsted Alvesson[1], Willem Stassen[2]

1 Department of Global Public Health, Karolinska Institutet, Stockholm, Sweden

2 Division of Emergency Medicine, University of Cape Town, Cape Town, South Africa

Corresponding author:

Helle Mölsted Alvesson

Karolinska Institutet, SE-171 77 Stockholm, Sweden

helle.molsted-alvesson@ki.se

Word Count: 4,300

For peer review only

ABSTRACT

Objectives: To determine emergency department physicians' perceptions regarding hospital companions being prohibited from accompanying the patient during COVID-19.

Methods: Two qualitative datasets were combined from the Western Cape, South Africa. Convenient sampling was used to recruit a total of eight physicians working full-time in the emergency department during COVID-19. Data collected included voice recordings, narrative interviewing, and semi-structured interviews. A reflexive thematic analysis was conducted and guided by the Normalisation Process Theory.

Results: The lack of physical companions provided an opportunity for physicians to assess and reflect on companion's role in efficient patient care. Physician's perceived that the COVID-19 restrictions illuminated that patient companions engaged in the emergency department as providers contributing to patient care, while simultaneously engaging as consumers detracting from physician's priorities and patient care. These restrictions prompted the physicians to consider how they understand their patients largely through the companions. When companions became virtual, the physicians were forced to shift how they perceive their patient, which included increased empathy.

For peer review only

Conclusion: The reflections of providers can feed into discussions of values within the health care system and can help explore the balance between medical and social safety, especially with companion restrictions still being practiced in some hospitals. These perceptions illuminate various tradeoffs physicians had to consider throughout the pandemic and may be used to improve companion policies when planning for the continuation of the COVID-19 pandemic and future disease outbreaks.

For peer review only

STRENGTHS AND LIMITATIONS OF THIS STUDY

- COVID-19 caused this study to pivot from purposive sampling to convenience sampling, potentially introducing a sampling bias affecting transferability and trustworthiness of the results.
- One professional base was included, potentially introducing a physician bias which can limit the transferability of this study.
- In-depth knowledge of one professional base was sampled at different time points between August 2020 to January 2022 providing a sufficient period to capture changed practices.
- By combining and comparing two datasets with different types of data, we were able to capture a wider range of perceptions, reinforcing our situational and reflexive data which has strengthened the quality and dependability of our analysis and results in relation to our research aim.

For peer review only

INTRODUCTION

Emergent conditions represent approximately 90% of global mortality (1). High functioning emergency departments (ED) are vital to alleviating this burden, however, the operations of EDs are influenced by the greater environment, social context and disease burden (1–3).

A variety of factors contribute to successful emergency departments. The World Health Organization (WHO) provides Essential Trauma Care Guidelines for a successful system and emphasizes the importance of the implementation of standard policies and protocols in acute care facilities (4). Policies and protocols take time to become established, and the tumultuous nature of COVID-19 posed challenges for maintaining and adapting standardised procedures (5). Additionally, EDs often face challenges with communication and mismanaged information; with health professionals in EDs spending 80% of their time engaging with communication processes,

For peer review only

which can include colleagues, management, patients and companions (6). In a high-risk, fast-paced environment like EDs, having established pathways of communication and information is critical to patient care, given that up to 12% of errors attributed to communication (6–8).

Prior to COVID-19, a prominent component of communication in EDs was conducted by patient companions; the literature highlights the strengths and challenges associated with this (9–13). Studies show companions can interfere with patient care by causing interruptions for the medical team, potentially leading to clinical errors, in addition, to increasing the stress on health care professionals when performing life-saving procedures (9,10). However, communication with companions is vital for gaining a quick medical history to inform effective care (9,10). Furthermore, the companion is a key component of support for both the patient and family resulting in greater patient motivation for recovery, reduced length of stay and less fear and anxiety for both the patient and family (9,11–13). The literature on companions pre-COVID show mixed findings on health professionals' perspective of patient companions and escorts, however, many studies find nurses are more in favour of familial presence than physicians, especially in emergency situations such as resuscitation (10,12).

For peer review only

During COVID-19, countries globally implemented policies that limited, if not completely restricted companions or escorts accompanying hospitalized patients to reduce transmission rates; inadvertently introducing various positive and negative outcomes (14,15). Studies have shown various impacts on the patient such as mental health challenges, reduced hygiene and nutritional status of patients (14–16). Companions and escorts experienced many emotional and mental consequences due to being separated including depression, fear, and anger (15). Lastly, providers were impacted by increasing demands for communication from families, and the stress of facilitating virtual communication (15,17). However, studies also reported positive impacts including less crowding, increased space for social distancing and reduced transmission for patients, companions and providers (18,19). Overall, most existing studies do not support universal restrictive policies in clinical settings during COVID-19, on the basis that the risks outweigh the benefits. The primary risks identified include psychological deficits to the patient and family, reduced patient health outcomes, trauma surrounding end of life care and increased stress for providers, in addition to the evidence on reduced transmission rates being limited in quality (14–16,20).

For peer review only

As of June 2022, South Africa has had approximately 4 million cases and 102,000 COVID-19 related deaths (21). The impacts of COVID-19 in South Africa, specifically the Western Cape are vast, such as increased mortality of COVID-19 and other diseases, delayed health-seeking behaviour and changes in injury presentation (22). EDs provide a unique setting to be impacted by COVID-19, and subsequent companion restrictions because they frequently serve vulnerable and marginalised populations (5). Additionally, the presentation and treatment of emergent conditions cannot be as easily controlled, scheduled or reorganised to fit within the new regulations COVID-19 places on the health system (5).

Although there is existing research on the broad COVID-19 impacts; the effects of companion restrictions throughout the COVID-19 pandemic have been minimally explored, especially outside the pediatric and palliative setting (15,16,19). In addition, the perspective of emergency health providers is rarely included. This study aims to determine emergency department physicians' perceptions regarding hospital companions and escorts being prohibited from accompanying the patient during COVID-19.

METHODS

For peer review only

South Africa is known for having some of the highest numbers of trauma in the world, in addition to experiencing a quadruple burden of disease (1,23,24). This study is specifically taking place in the Western Cape, South Africa because a high proportion of the countries' emergent conditions and diseases present in EDs here (23). It was also the first province within South Africa to experience a COVID-19 surge and has maintained a relatively high rate of cases and deaths, with approximately 21% of cases and 22% of COVID-19 related deaths in the country (25). Specifically, COVID-19 affected practices that contribute to successful care in South African EDs, such as companion restrictions, changing protocols, structurally splitting the ED into COVID-19 likely and COVID-19 unlikely, resource distribution, reduction of blood supply, shortage of PPE and the redistribution of health professionals leaving many departments with insufficient human resources (26,27). The high burden of emergent conditions and COVID-19 makes it a unique and appropriate setting to evaluate the impacts of COVID-19, on their already burdened emergency system.

Study Design

The study utilised two different qualitative datasets. Dataset I included data from the end of the first wave in August to October 2020 and explored emergency medical personnel's lived

For peer review only

experiences with the COVID-19 pandemic (28). Dataset II was collected in the second and third wave, from February 2021 to January 2022, and included semi-structured interviews of physicians' perceptions on how COVID-19 affected emergency care.

Sampling and Recruitment

Both datasets included in-hospital emergency personnel in the Western Cape. Convenient sampling was utilised, and emergency medical joint staff were contacted using their registered email addresses in the UCT staff repository. Staff were asked to distribute the study invitation to their employees for equal access. Subsequent participants were collected through snowball sampling. Our proposed sample size was 12 participants, but our priority was reaching information saturation which was assessed throughout the data collection process. Our sample concluded with eight participants, who worked full-time in the emergency department throughout COVID-19, from six different hospitals.

The mean age of participants was 35 years old, with 9 years of medical experience. Demographic information was gained through a verbal questionnaire. Their sample characteristics are included in table 1 while maintaining anonymity (Table 1).

Table 1 - Participant ID Codes

For peer review only

ID Code	Self-Identified Gender	Job Title	Years in profession (since qualification)
ID1	Male	Medical officer	9
ID2	Female	Consultant	11
ID3	Female	Consultant	5
ID4	Female	Medical officer	11
ID5	Female	Registrar	6
ID6	Male	Medical officer	8
ID7	Male	Registrar	9
ID8	Female	Registrar	8

For peer review only

Data Collection

This study aimed to answer two questions (1) which practices in the emergency department do the physicians perceive as being changed by the restrictions on companions/escorts throughout COVID-19 and (2) how have the restrictions impacted how physicians conceptualize the role of companions in patient care? Dataset I included WhatsApp voice recordings and one-on-one online interviews, conducted in English or Afrikaans. Only physicians from this dataset were included. Voice recordings ranged from 17 seconds to 30 minutes, and interviews ranged from 40-60 minutes. In dataset II, a semi-structured interview guide (Appendix 1) was used in all interviews and was inspired by the Normalisation Process Theory (NPT). The interview guide was piloted for clarity with an emergency physician in

Sweden and was adjusted accordingly. The interviews were conducted online, in English and ranged from 40-60 minutes.

Ethical approval was obtained from the University of Cape Town's Faculty of Health Sciences Human Research Ethics Committee (HREC ref. 052/2021) (Appendix 2). The ethical application for dataset I (HREC ref. 386/2020) included approval for the anonymised data to be reanalysed for similar research questions (Appendix 3). All participants were provided with an information and consent letter, and they were able to ask any questions before signing (Appendix 4,5). Further approval was sought from the Provincial Department of Health and the hospitals for participants to feel safe participating and have no repercussions in their workplace.

Patient and Public Involvement

We had no patient/public involvement.

Analysis

The study aim and questions are inspired by Reflexive Monitoring which has been identified as an important feature in qualitative improvements, as it highlights how people assess the impacts of new practices on themselves and others (29). In our context, we are examining how physicians assess companion restrictions in their personal practice, along with the appraisal

For peer review only

of their colleagues, companions, and the patient. Reflexive Monitoring is a part of the Normalisation Process Theory (NPT), which focuses on the processes behind changes in thinking, practices, policies and organisation in healthcare settings, specifically resource-constrained contexts (29).

(Figure 1).

The data from both studies were subjected to an inductive, reflexive thematic analysis using the six steps as described by Braun and Clarke (30). NVivo V.12 Pro (QSR International Pty Ltd, Australia) was used to develop the main categories and themes. Transcripts were initially coded by one data collector and a list of latent codes was developed. After the interviews were coded, subcategories were discussed in relation to emerging themes and were narrowed down to identify the main theme(s). These steps were done as an iterative process, where the codes and emerging themes were continuously reviewed amongst the research team, to help achieve triangulation and comprehensive analysis (31). Throughout the analysis, the data was reviewed in relation to reflexive monitoring in the NPT, however, this was used to reinforce, not determine, or limit the themes. Anonymity was prioritized in this study and each participant was

For peer review only

given an ID code to ensure their information was not linked to their responses or quotations. Pseudonyms were used in the transcriptions

Reflexivity

The research group was made up of an interdisciplinary team, all with experience with qualitative studies in Cape Town, in addition to one of our primary investigators having extensive research experience within emergency medicine. Furthermore, the interviewer's position and relationship with participants can impact the comfort and openness of participants (32). The interviewer was a master's student of public health, with no ties to the hospitals that the physicians worked at potentially contributed to building trust and the physicians feeling more comfortable to share information about their workplace.

RESULTS

During COVID-19, the restriction was implemented that no companion was allowed to accompany the patient upon arrival to the ED to ensure the safety of all parties by reducing the risk of COVID-19 transmission (27). In the absence of physical companions, practices surrounding patient support, communication with companions and

For peer review only

gaining collateral information were disrupted, as companions became virtual. Based on the perceptions of the physicians, this change illuminated the different roles physical companions hold in patient care as both providers, contributing to patient care, and consumers of care, potentially detracting from the patient in the ED (Table 2).

Table 2 – Results Table

**Theme**	**Reconceptualising Companions in the Emergency Department**		
**Category**	Virtual Companions		Finding Balance
	Companions as Providers	Companions as Consumers	
**Codes**	Patient support<br>Collateral information<br>Nature of communication<br>Provider empathy	Interruptions<br>Crowding<br>Call volume<br>Time consumption	Visiting hours<br>Technology<br>Contact sheets

Companions as Providers in the ED

Pre-COVID, physicians relied on patients being physically accompanied to the ED to communicate with companions. Physicians' acknowledged companions play an important role for both the patient and the provider in the ED. When companions were not physically present, it often resulted in the patient being dependent on the health provider to communicate with the family.

For peer review only

"A lot of them [patients] would not necessarily have a cell phone or a charged cell phone, or data to contact the families. And so, there were a lot of them that were very anxious and that definitely made it difficult for us, because it's important for them to also have communication with their families, especially when they're sick and their families need to know what's happening."- ID1

The physicians expressed that the prominent negative aspect of not having physical companions were the challenges associated with obtaining collateral patient information relevant for clinical care including pre-existing conditions, family history and medications. They viewed this as problematic to their ability to provide care.

"Having a physical escort there is often the only way to get collateral about a patient, and especially during COVID, so much about your decision about the level of care, and level of intervention was based on a patient's baseline … when these people didn't answer their phones, or you didn't have those numbers, it was literally impossible to make these kinds of decisions." – ID5

Physicians expressed that getting a hold of the companions was often time-consuming and occasionally, they never could get a hold of them, meaning they were having to make decisions regarding care without that critical information. Physicians also reflected on companion presence pre-COVID, and identified the physical presence resulted in higher quality, and individualized care planning, compared to trying to get a hold of companions virtually.

For peer review only

“This meant a lot more digging in folders looking for ID numbers, trying to contact family members outside of the hospital, phones that weren't answered, that really made an impact on how we manage patients and it also delayed turnaround time. Which meant we had patients boarding a lot longer in an emergency unit trying to figure out what is going on to form some sort of disposition plan for a patient.”- ID6

Companions as Consumers in the ED

Although physicians acknowledged various ways that consumers contribute and enhance practices in the ED, the shift to virtual companions also illuminated ways that physical companions interfered with different practices. One reason physicians expressed a preference for virtual companions introduced during COVID-19 was because they viewed patient treatment as their primary task and having virtual companions increased the efficiency of their clinical tasks. Specifically, due to fewer interruptions and less crowding while treating the patient, they felt they were able to prioritize patient care with fewer mistakes.

“Before there were two visiting times per day, an hour-long visiting time, which means that every patient in the unit could have two people within that hour, which literally triples the amount of people in the room. So, it's also just getting in the way, it's two hours of the day where you can’t do anything because there's no point in being there.” – ID5

For peer review only

"So not having those people to kind of judge you for what was going on definitely took off the stress of the work to some degree, because you could kind of get on with what you needed to do." - ID8

To compensate for physical presence, EDs implemented various practices such as contact sheets upon arrival, tick sheets, involving other departments such as psychology or having a dedicated health provider conducting all the calls to families. Physicians expressed they were able to communicate suggestions for different solutions to the consultant level, who worked with management to implement different systems. However, physicians' felt these methods frequently resulted in calls not being conducted, or the physician completing the call themselves. It also created increased unrest amongst the virtual companions resulting in a higher number of calls to the ED overwhelming their phone system; a phone system designated for emergent referrals. The in-person interruptions that occurred pre-COVID, shifted to the phone, especially with many companions wanting to speak to the physician directly, not other providers. The time-consuming nature of these changes resulted in less time the physician was available for tasks related to patient care.

For peer review only

"The phone calls to the hospital were very difficult to handle because sometimes it'd be 22 family members of the same patient who all wanted a detailed update from the doctor. Each of which identified themselves as the family spokesperson." – ID3

Separate from the frequency of communication, physicians expressed the nature of communication with companions shifted throughout COVID-19 due to the severity and rapid changes in patient conditions. Physicians acknowledged having companions be virtual exacerbated how hard these conversations were. In addition to changing communication with companions, it impacted how the provider interacted with the patient including being more selective with their words and increasing compassion and empathy.

"it's probably one of the hardest things I've ever had to do to phone so many families and tell them that their family members died or was going to die or wasn't going to make it. And so, I really had to dig deep because it wasn't, as hard as it is for me, it's obviously harder for the patients' families. I think I definitely learned to kind of find the compassion even when you're tired and exhausted." - ID8

Finding Balance for Companion Engagement

Beyond the time-consuming nature of updating virtual companions, physicians were able to identify both benefits and challenges associated with virtual companions, throughout COVID-19 and within the context of the ED in general. They expressed that

For peer review only

finding a hybrid of pre-COVID practices and COVID-19 restrictions moving forward is necessary for providers, patients, and companions. Physicians offered up different suggestions such as increasing the capacity to facilitate video calling, limiting the number of companions at the bedside, allowing one designated patient spokesperson at the bedside, reducing the length of visiting hours and having companions fill in up to date contact information when dropping off patients.

"We need to find something that works a bit better for families, but also allows us to actually see our patients timeously and not have patients waiting for 12 hours to see a doctor, those kinds of things." – ID3

"We sometimes easily lack the human element in the emergency department when pressed for beds and patients and just chaos that is generally in the ED…. There was across the board, from all my colleagues, a much more human aspect to that kind of [patient] interaction, than there were before."-1D7

Physicians discussed that COVID-19 changes, specifically patients having no companions, illuminated lacking practices in EDs that balanced humanity factors with the dynamic nature of emergency medicine. Physicians acknowledged finding a way for these factors to co-exist is an important lesson to bring forward throughout and post-COVID-19.

DISCUSSION

For peer review only

COVID-19 caused many formal and informal changes that providers were juggling in conjunction with companion restrictions. Formal changes included structurally splitting the ED, different clinical protocols and prohibiting patient companions, in addition to informal changes such as disrupting tacit knowledge, provider decision-making and communication adaptations. Physicians were able to provide insight from their perspective into the impacts companion restrictions had on the ED, amidst all the other changes, which can help inform future change and preparedness.

Physicians were faced with a new scenario of no companions accompanying the patient, yielding a paradox regarding the efficiency and quality of patient care with companions engaging as both providers and consumers in the ED. How emergency physicians perceive the lacking physical presence of companions emerges as an understudied area, specifically in EDs and should be incorporated into the greater debate on patient companions. Existing literature primarily focuses on companions from a patient-centered care perspective, as companions are found to contribute to feelings of patient agency, safety and improved health outcomes among others (11–13). Totten et al., (2014) found that 78% of patients in the ED felt their companion was important to

For peer review only

their care for social support and advocacy (13). Although there are no official existing guidelines or recommendations for companions in the ED, there is a large body of literature in support of companions for different health care settings such as maternal and ICU (33,34). For example, the WHO has issued recommendations for companions of choice during labour and childbirth and argues having a companion they trust contributed to women feeling safe, strong and lead to improved maternal and perinatal outcomes. One of the main factors they contribute this to are that companions bridge communication between the healthcare professionals and women (34).

Overall, physicians acknowledged benefits and challenges with virtual companions, and expressed the desire to find a balance moving forward between the policies and practices pre-COVID and during COVID-19. Iness et al. and Herbst et al. conducted studies that include the physician perspective in the ICU during COVID-19 and found physicians perceived virtual companions and telecommunication to increase their workload and limited effective and empathetic communication (20,35). This aligns with our findings that physicians noticed virtual companions to be more time-consuming, however, there are minimal studies that include the physician perspective, and none

For peer review only

conducted specifically in the ED. Therefore, more research is needed to situate our specific findings, to propose comprehensive and sustainable solutions moving forward.

In addition to companion restrictions, physicians expressed that COVID-19 patients were not moved to isolation facilities but were streamlined within the hospital to different “COVID likely” and “COVID unlikely” areas in EDs based on their respiratory symptoms. The literature from other recent disease outbreaks such as SARS and Ebola, predominantly supports physical companion presence on the basis that it yields greater benefits to the patient than risks (36). It’s argued that restricting companions can discourage patients from seeking care, which can undermine infection control efforts, as found in the Ebola response (37). When positioning our findings within literature on disease outbreaks, it identifies that the hospital and health system has insufficiently applied lessons learned about companions from previous outbreaks. There needs to be increased incorporation of companion restriction research for future emergency preparedness and response planning.

Participating physicians perceived their primary role as a provider to be treating the patient and that having virtual companions was beneficial as it reduced interruptions,

For peer review only

errors, and stress. Results are quite divided from the limited ED literature on health providers' views of companions under normal circumstances. Timmermans and Meyers et al., propose that health professionals perceive companions as a hindrance to the process of saving a patient's life (38). Generally, physicians were more worried than other health professions about the presence of companions including crowding and interruptions, and according to Readly and Hood, 61% of health professionals felt they would have increased anxiety and stress in life-saving procedures with companions' present (38). The alignment of our findings with the literature illuminates the importance of including physician input when implementing practices and policies, as it impacts physician performance and subsequently patient care and safety. Further research needs to be conducted to find a balance respecting the physicians' perspective, while protecting patient-centred care.

Physicians perceived companions as important for gaining collateral information about the patient. In the ED focused literature, physicians primarily did not perceive companions to be associated with patient care, even though they do identify companions play an important role in providing critical health information as the patient's

For peer review only

spokesperson (38). However, there is a knowledge gap in existing research, for the effects and potential added inefficiency that is associated with physical companions being switched to virtual communication. This gap could be attributed to the fact that the majority of existing literature is not occurring amongst provider populations that have experienced companion restrictions before, and therefore, have never actively had to consider how care and efficiency would shift when the companions switch to being virtual.

COVID-19 provided an opportunity for physicians to engage with reflexive monitoring as they appraise the role of companions, now having a prolonged experience without them. Moving forward, there is an opportunity for further research into if these experiences have led to changes in how physicians perceive companions as an important component of care.

Strengths and Limitations

This study obtained in-depth knowledge of one professional base sampled at different time points between August 2020 to January 2022 which provides a sufficient period to capture changed practices.

For peer review only

This study faced challenges with recruitment attributed to the delays caused by the COVID-19 vaccine roll-out, which put research project approvals on hold. Due to the extenuating circumstances, this study had to pivot from purposive sampling and utilised convenience sampling, which can introduce a sampling bias caused by lack of equal access to participation affecting the transferability and trustworthiness of the results (39). However, the sample includes experienced physicians who were able to compare practices before and during the pandemic. However, since COVID-19 is ongoing, we are not able to exhaustively capture how the physicians continue to perceive the changes.

Due to the sampling and recruitment limitations, we chose to maximize our understanding of the perceptions in one professional base. The physician bias limits the transferability of this study.

This study combined two qualitative datasets that utilised different interview methodology. This potentially posed challenges for analysis because it introduced the potential for bias when coding dataset I which did not use an interview guide, as the coder may be inclined to look for codes or themes that align with the main topics from the dataset II interview guide. This bias was reduced by continuously discussing the data

For peer review only

amongst the research team to ensure fair coding. However, both studies focused on gathering perceptions from in-hospital emergency physicians; by combining these two datasets with different types of data, we were able to capture a wider range of perceptions. In the voice recordings, physicians were able to share their perceptions in real-time pre and post shift, allowing relevant situational information to be shared, that might have been forgotten by the time of a follow-up interview. We were able to compare both datasets reinforcing our situational and reflexive data which has strengthened the quality and dependability of our analysis and results in relation to our research aim (31).

Implications of the Study

Learning from physicians' experiences and perspectives can help create a more holistic understanding of the contribution the companion has in the ED. This can inform companion policies that will promote preparedness, patient-centred care, and improved health outcomes; in both the everyday context and in preparing for the continuation of the COVID-19 pandemic and other future disease outbreaks. It also provides a platform for future research to increase emergency preparedness, specifically studies that include

For peer review only

more professional bases to gain a wider perspective of the companion restrictions, in addition, to follow up studies once COVID-19 has settled to learn how these changes were normalised.

CONCLUSION

Physicians were introduced to the benefits and challenges associated with virtual companions. They felt the benefits of reduced crowding and interruptions for bedside care, and the negatives associated with the lack of collateral information and time-consuming nature of communication with companions virtually. Overall, physicians expressed their hope that policies achieving a hybrid of pre-COVID and COVID practices will be sustained moving forward, including suggestions of contact information sheets, limiting numbers of bedside companions, and increasing access to video technology. By learning from their perceptions, there is an opportunity to better prepare the ED and providers for the next pandemic or crisis, that may affect companion policies, to maintain the highest level of patient centered care.

Acknowledgements

The authors wish to thank all the physicians who participated and shared their experiences.

For peer review only

Figure Legend

Figure 1.1 – visual representation of the Normalisation Process Theory.

Contributorship Statement

All authors contributed to the conception and design of the study. LW was the primary contributor to data collection, data analysis and interpretation, paper writing, revisions and submission. HMA contributed to the onoging qualitative methodology design, data analysis and interpretation, draft and final submission revisions. WS contributed to sampling and recruitment, draft and final paper revisions, data sharing and providing the final approval for submission. All authors agree to be accountable for their work. We would like to acknowledge Elzarie Theron and Helena Erasmus for their contribution with the data collection from dataset I.

Competing Interests

The authors have declared that no conflict of interests exist.

Funding

None to report.

Data Sharing Agreement

Within the transcripts, physicians discuss personal and potentially identifying experiences from their workplace. The informed consent that all physicians signed promised full anonymity, therefore making the full data set publicly available would potentially breach their privacy. Following data requests, transcripts will be reviewed for any potential identifying information and will only be made available to researchers who sign a data sharing agreement. Data requests may be sent to Willem Stassen at willem.stassen@uct.ac.za.

Ethics Approval Statement

This study involves human participants and was approved by University of Cape Town's Faculty of Health Sciences Human Research Ethics Committee (HREC ref. 052/2021).

For peer review only

References

1. Chang CY, Abujaber S, Reynolds TA, Camargo CA, Obermeyer Z. Burden of emergency conditions and emergency care usage: New estimates from 40 countries. Emergency Medicine Journal. 2016 Nov 1;33(11):794–800.

2. Gunning AC, Lansink KWW, Van Wessem KJP, Balogh ZJ, Rivara FP, Maier R V., et al. Demographic Patterns and Outcomes of Patients in Level i Trauma Centers in Three International Trauma Systems. World J Surg. 2015 Nov 1;39(11):2677–84.

3. Reynolds TA, Stewart B, Drewett I, Salerno S, Sawe HR, Toroyan T, et al. The Impact of Trauma Care Systems in Low-and Middle-Income Countries. Annu Rev Public Health. 2017;38:507–39.

4. Word Health Organization. Guidelines for Essential Trauma Care. 2004.

5. Haut ER, Leeds IL, Livingston DH. The Effect on Trauma Care Secondary to the COVID-19 Pandemic. Ann Surg. 2020 Sep;272(3):e204–7.

6. Creswick N, Westbrook JI, Braithwaite J. Understanding communication networks in the emergency department. BMC Health Serv Res. 2009 Dec 31;9(1):247.

7. Pun JKH, Matthiessen CMIM, Murray KA, Slade D. Factors affecting communication in emergency departments: doctors and nurses' perceptions of communication in a trilingual ED in Hong Kong. Int J Emerg Med. 2015 Dec 1;8(1):1–12.

8. Reddy MC, Paul SA, Abraham J, Mcneese M, Deflitch C, Yen J. Challenges to effective crisis management: Using information and communication technologies to coordinate emergency medical services and emergency department teams. 2008;

9. Kilner E, Sheppard LA. The role of teamwork and communication in the emergency department: A systematic review. Int Emerg Nurs. 2010 Jul 1;18(3):127–37.

10. Porter J, Cooper SJ, Sellick K. Attitudes, implementation and practice of family presence during resuscitation (FPDR): A quantitative literature review. Int Emerg Nurs. 2013 Jan 1;21(1):26–34.

11. Berwick D, Downey A, Cornett E, Sector C on MTCLHS and IT to the C, Policy B on HS, Populations B on the H of S, et al. Delivering Patient-Centered Trauma Care. 2016 Sep 12;

For peer review only

12. Olding M, McMillan SE, Reeves S, Schmitt MH, Puntillo K, Kitto S. Patient and family involvement in adult critical and intensive care settings: a scoping review. Health Expectations. 2016 Dec 7;19(6):1183–202.

13. Totten VY, Bryant TK, Chandar AK, Hoch WB, Hunter SL, Patel NJ, et al. Perspectives on Visitors in the Emergency Department: Their Role and Importance. J Emerg Med. 2014 Jan 1;46(1):113–9.

14. Jaswaney R, Davis A, Cadigan RJ, Waltz M, Brassfield ER, Forcier B, et al. Hospital Policies during COVID-19: An Analysis of Visitor Restrictions. Journal of Public Health Management and Practice. 2021;

15. Hugelius K, Harada N, Marutani M. Consequences of visiting restrictions during the COVID-19 pandemic: An integrative review. Int J Nurs Stud [Internet]. 2021 Sep 1 [cited 2022 Apr 24];121:104000. Available from: /pmc/articles/PMC8196532/

16. Lo AX, Wedel LK, Liu SW, Wongtangman T, Thatphet P, Santangelo I, et al. COVID-19 hospital and emergency department visitor policies in the United States: Impact on persons with cognitive or physical impairment or receiving end-of-life care. J Am Coll Emerg Physicians Open [Internet]. 2022 Feb [cited 2022 Apr 24];3(1). Available from: /pmc/articles/PMC8776041/

17. Fino E, Fino V, Bonfrate I, Russo PM, Mazzetti M. Helping patients connect remotely with their loved ones modulates distress in healthcare workers: a tend-and-befriend hypothesis for COVID-19 front liners. Eur J Psychotraumatol. 2021;12(1).

18. Wendlandt B, Kime M, Carson S. The impact of family visitor restrictions on healthcare workers in the ICU during the COVID-19 pandemic. Intensive Crit Care Nurs. 2022 Feb 1;68:103123.

19. Virani AK, Puls HT, Mitsos R, Longstaff H, Goldman RD, Lantos JD. Benefits and risks of visitor restrictions for hospitalized children during the COVID pandemic. Pediatrics [Internet]. 2020 Aug 1 [cited 2022 Apr 24];146(2). Available from: /pediatrics/article/146/2/e2020000786/36916/Benefits-and-Risks-of-Visitor-Restrictions-for

20. Iness AN, Abaricia JO, Sawadogo W, Iness CM, Duesberg M, Cyrus J, et al. The effect of hospital visitor policies on patients, their visitors, and healthcare providers during the COVID-19 pandemic: a systematic review. Elsevier Public Health Emergency Collection . 2022 Apr 23;

For peer review only

21. COVID-19 South African Online Portal. COVID-19 Corona Virus South African Resource Portal. 2021.
22. Mbunge E. Effects of COVID-19 in South African health system and society: An explanatory study. Diabetes and Metabolic Syndrome: Clinical Research and Reviews. 2020 Nov 1;14(6):1809–14.
23. Hanewinckel R, Jongman HP, Wallis LA, Mulligan TM. Emergency medicine in Paarl, South Africa: A cross-sectional descriptive study. Int J Emerg Med. 2010;3(3).
24. Mayosi BM, Flisher AJ, Lalloo UG, Sitas F, Tollman SM, Bradshaw D. The burden of non-communicable diseases in South Africa. Lancet. 2009;374(9693):934–47.
25. Western Cape Government. Covid-19 Dashboard | Covid-19 Response [Internet]. 2022 [cited 2022 Apr 26]. Available from: https://coronavirus.westerncape.gov.za/covid-19-dashboard
26. Nyasulu J, Pandya H. The effects of coronavirus disease 2019 pandemic on the South African health system: A call to maintain essential health services. Afr J Prim Health Care Fam Med. 2020;12(1).
27. Mendelson M, Booyens L, Boutall A, Cairncross L, Calligaro G, Dave JA, et al. The mechanics of setting up a COVID-19 response: Experiences of the COVID-19 epidemic from Groote Schuur Hospital, Cape Town, South Africa. South African Medical Journal. 2020;110(10).
28. Theron E, Erasmus HC, Wylie C, Khan W, Geduld H, Stassen W. The lived experiences of emergency care personnel in the Western Cape, South Africa during the COVID-19 pandemic: A longitudinal hermeneutic phenomenological study. African Journal of Emergency Medicine [Internet]. 2022 Dec 1 [cited 2022 Nov 30];12(4):410. Available from: /pmc/articles/PMC9420724/
29. Huddlestone L, Turner J, Eborall H, Hudson N, Davies M, Martin G. Application of normalisation process theory in understanding implementation processes in primary care settings in the UK: A systematic review. Vol. 21, BMC Family Practice. BioMed Central Ltd.; 2020.
30. Braun V, Clarke V. Using thematic analysis in psychology. Qual Res Psychol. 2006;3(2):77–101.

For peer review only

31. Schutt R. Investigating the Social World - The Process and Practice of Research [Internet]. 9th edition. 2018 [cited 2021 May 5]. p. 728. Available from: https://us.sagepub.com/en-us/nam/investigating-the-social-world/book254625

32. Berger R. Now I see it, now I don't: researcher's position and reflexivity in qualitative research: http://dx.doi.org/101177/1468794112468475. 2013 Jan 3;15(2):219–34.

33. Williams CMA. The identification of family members' contribution to patients' care in the intensive care unit: a naturalistic inquiry. Nurs Crit Care. 2005;10(1):6–14.

34. Companion of choice during labour and childbirth for improved quality of care.

35. Herbst N, Kuntz J. Impact of COVID-19 Visitation Restrictions on ICU Communication, Provider Attitudes and Burnout. Chest. 2021;160(4).

36. Voo TC, Lederman Z, Kaur S. Patient Isolation during Infectious Disease Outbreaks: Arguments for Physical Family Presence. Public Health Ethics. 2020 Jul 1;13(2):133–42.

37. Voo TC, Senguttuvan M, Tam CC. Family Presence for Patients and Separated Relatives During COVID-19: Physical, Virtual, and Surrogate. J Bioeth Inq. 2020;1.

38. Axelsson ÅB, Zettergren M, Axelsson C. Good and bad experiences of family presence during acute care and resuscitation. What makes the difference? Vol. 4, European Journal of Cardiovascular Nursing. Eur J Cardiovasc Nurs; 2005. p. 161–9.

39. Robinson OC. Sampling in Interview-Based Qualitative Research: A Theoretical and Practical Guide. Qual Res Psychol. 2014 Jan;11(1):25–41.

For peer review only

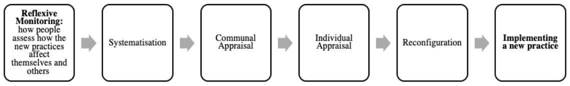

For peer review only

APPENDIX 1: Interview Guide

Thank you for accepting to take part in this interview. Our goal is to learn about your perspective of the changing processes in your trauma care facility during COVID-19, with a particular focus on any changes that have occurred due to no hospital visitors. The interviews will be conducted by Lauren Wiebe, a Master's student from Canada in Public Health Sciences at Karolinska Institutet under the supervision of Dr Helle Alvesson and Dr Willem Stassen.

Experience

A. Prior to COVID-19, what was your role in this unit, and can you describe a bit about your work? For example, what kind of tasks did you do? Who in the unit did you work with? What kind of patients did you see?
B. Before COVID-19, approximately how many patients did you see in a day? How many do you see now? Approximately what month do you feel this changed?
C. Besides COVID-19, during your time working at this hospital have you ever had to work through a secondary emergency before e.g. epidemic, natural disasters, conflict? Have you had this type of experience at another health facility?

Changing practices during COVID-19

A. Can you list some of the practices that have changed in your unit since COVID-19?

→ *The answers from this list can be used as prompts for the below questions. Examples: intake/triage process, referral system, caregiver presence, health seeking behaviour, teamwork among the unit, resource distribution*

B. Please describe what aspects of these changes you value? What aspects do you dislike?
C. Please describe how you learned to adapt to the changing practices? For example, access to training, protocol documents or team meetings.

Making sense of the changes

A. How do these changes compare to your practices before? Can you please provide me with an example?
B. Do you feel like the changes that have occurred are important? If yes, please describe.
C. Do you think your colleagues share an understanding of these changes and their importance? If yes, please describe what influenced this shared understanding? If no, please describe why there are perceived differences?

Participating in the changes

A. In your opinion, do the staff in your unit all equally participate in these changes? If no, what do you think are the main reasons people are not participating in these changes?
B. Are there individuals in the unit who are promoting and supporting the team in these changes? For example, a management team, chief doctor etc.
    a. If yes, please describe the ways they promote/support the unit during COVID?
    b. If yes, did they hold these leadership positions prior to COVID-19?
    c. If there is no central promotor/enforcer, what do you think motivates the staff to participate?
C. Do you feel the new practices are sustainable moving forward? If yes, please explain.

For peer review only

Operationalization of the changes

A. Do you think all staff members in your unit are equally affected by the changes from COVID-19?
B. Since COVID-19, have you seen a change in how the resources are distributed?
C. How have relationships and interactions in your job changed with the changing practices?

Monitoring and assessment of the changes

A. In your opinion have these new practices been effective? Has anything improved from how they were practiced pre-covid? If so, can you give me an example?
B. Please describe how these changes have affected how the medical team works together?
C. How do you feel these changes have affected you individually and your job tasks?
D. Among these changes, what practices do you think could be improved? If so, can you give me an example?

**If not previously discussed-* We would like to ask you a few more questions specifically about the changes you experienced with no caregivers being able to accompany the patient into the health facility.

A. In general, did you notice any changes to care with no caregivers/family being able to accompany the patient?
B. How did the restrictions on family members affect your communication with the patient?
    a. How did you communicate the health news to the families?
    b. How did it influence how you communicated with your colleagues?
C. How did you feel about no caregivers/family members being allowed?
D. What do you think the patients felt having no caregiver with them?
E. In your opinion, what do you feel could have made this better?

Final Questions

A. Are there any other comments you would like to share related to your personal experience working with COVID-19 that have not been addressed in this interview?
B. Do you have any final questions for me?

Questionnaire to be completed before or after the interview:

A. What is your age?
B. What is your identified gender?
C. What is your profession and specialization?
D. How many years have you been working in the health profession?
E. How many years have you been working at this hospital?
F. During the COVID-19 outbreak, were you working part-time or full-time?

For peer review only

APPENDIX 2: Ethical Approval Study II

UNIVERSITY OF CAPE TOWN
Faculty of Health Sciences
Human Research Ethics Committee

Room G50- Old Main Building
Groote Schuur Hospital
Observatory 7925
Telephone [021] 406 6492
Email: hrec-enquiries@uct.ac.za
Website: www.health.uct.ac.za/fhs/research/humanethics/forms

23 February 2021

HREC REF: 052/2021

Dr W Stassen
Division of Emergency Medicine
F51, OMB
Email: willem.stassen@uct.ac.za

Dear Dr Stassen

PROJECT TITLE: THE PERCEPTIONS AND EXPERIENCES OF EMERGENCY MEDICAL STAFF ON THE IMPACT OF COVID-19 ON TRAUMA EMERGENCY CARE SERVICE PROVISION IN THE WESTERN CAPE PROVINCE OF SOUTH AFRICA

Thank you for your response letter, addressing the issues raised by the Faculty of Health Sciences Human Research Ethics Committee (HREC).

It is a pleasure to inform you that the HREC has **formally approved** the above-mentioned study, subject to the following: -

1. There is one outstanding issue. The section on Reflexivity (which seems to be in the wrong place, there is a section on the one visiting master's student. (See below)
   Reflexivity
   Lauren Wiebe has a bachelor's degree in Public Health and Political Science from Vancouver, Canada and is currently a Master's in Public Health Sciences student at Karolinska Institutet. The majority of her experience surrounds public health policy for mental health and infectious diseases for populations in Canada. She has no direct experience working in or with trauma healthcare, reducing preconceptions. However, preconceptions around the role of health workers during the pandemic are that changes have occurred within the trauma unit, potentially in the areas such as teamwork, patient care, protocols and resource distribution.
   Can the researchers please tidy this up and put reflexivity together with the section on credibility and trustworthiness; and put the detail about Lauren in the appropriate place.

This approval is subject to strict adherence to the HREC recommendations regarding research involving human participants during COVID -19, dated 17 March 2020 & 06 July 2020.

Approval is granted for one year until the 28 February 2022.

Please submit a progress form, using the standardised Annual Report Form if the study continues beyond the approval period. Please submit a Standard Closure form if the study is completed within the approval period.
(Forms can be found on our website: www.health.uct.ac.za/fhs/research/humanethics/forms)

Please quote the HREC REF 052/2021 in all your correspondence.

Please note that the ongoing ethical conduct of the study remains the responsibility of the principal investigator.

HREC/REF 052/2021sa

APPENDIX 3: Ethical Approval Dataset I

UNIVERSITY OF CAPE TOWN
Faculty of Health Sciences
Human Research Ethics Committee

Room G50- Old Main Building
Groote Schuur Hospital
Observatory 7925
Telephone [021] 406 6492
Email: hrec-enquiries@uct.ac.za
Website: www.health.uct.ac.za/fhs/research/humanethics/forms

06 August 2020

HREC REF: 386/2020

Dr W Stassen
Division of Emergency Medicine
c/o Ms Vathiswa Mzamo
F-51, OMB
Email: willem.stassen@uct.ac.za

Dear Dr Stassen

PROJECT TITLE: LIVED EXPERIENCES OF EMERGENCY CARE PERSONNEL IN THE WESTERN CAPE, SOUTH AFRICA DURING THE COVID-19 PANDEMIC: A LONGITUDINAL HERMENEUTIC PHENOMENOLOGICAL STUDY

Thank you for submitting your study to the Faculty of Health Sciences Human Research Ethics Committee (HREC) for review.

It is a pleasure to inform you that the HREC has **formally approved** the above-mentioned study.

This approval is subject to strict adherence to the HREC recommendations regarding research involving human participants during COVID -19, dated 17 March 2020 & 06 July 2020.

Approval is granted for one year until the 30 August 2021.

Please submit a progress form, using the standardised Annual Report Form if the study continues beyond the approval period. Please submit a Standard Closure form if the study is completed within the approval period.
(Forms can be found on our website: www.health.uct.ac.za/fhs/research/humanethics/forms)

Please quote the HREC REF in all your correspondence.

Please note that the ongoing ethical conduct of the study remains the responsibility of the principal investigator.

Please note that for all studies approved by the HREC, the principal investigator **must** obtain appropriate institutional approval, where necessary, before the research may occur.

Yours sincerely

PROFESSOR M BLOCKMAN
CHAIRPERSON, FHS HUMAN RESEARCH ETHICS COMMITTEE

HREC 386/2020sa

APPENDIX 4: Consent Letter Study II

PART 1: INFORMED CONSENT FORM

The perceptions and experiences of emergency medical staff on the impact of COVID-19 on trauma emergency care service provision in the Western Cape province of South Africa

Investigators and affiliations
Dr Willem Stassen, Div. Emergency Medicine, University of Cape Town
Prof Helle M. Alvesson, Dept. Global Public Health, Karolinska Institutet
Ms Elzarie Theron, Div. Emergency Medicine, University of Cape Town
Ms Lauren Wiebe, Dept. Global Public Health, Karolinska Institutet

You are invited to participate in a study conducted by the Division of Emergency Medicine at the University of Cape Town and the Department of Global Public Health at the Karolinska Institutet, Sweden.

COVID-19 was declared a global pandemic in March 2020 following a cluster of pneumonia cases in Wuhan, China. Caused by severe acute respiratory syndrome Coronavirus -2 (SARS-CoV-2), COVID-19 continues to spread across the globe. The purpose of this study is to understand the perceptions of emergency care personnel in the Western Cape on the changes in care provision due to COVID-19. We aim to learn from your perspective including, what trauma care processes and practices changed due to COVID-19 and the various ways you had to adjust, specifically inquiring about your perceptions regarding no hospital visitors and escorts. Understanding this perspective can mitigate operational consequences of the COVID-19 pandemic and in the planning for future pandemics. You were selected to participate in this study because you are working clinically on the frontline during the COVID-19 pandemic. Doctors and nurses regardless of baseline qualification are eligible. We aim to recruit a maximum of 20 frontline clinical providers in this study.

Voluntary Participation
Whether you decide to participate in the study or not is entirely your choice and voluntary. There will be no consequences to you if you decide not to participate. You may also decide to change your mind and may withdraw from participating, even if you agreed to it at an earlier stage.

Description of the Process
The interviews will take place over a period of 1-3 months in February-April 2021 and we will require your participation at one session within this period. If you choose to participate in this study, your interview session will take approximately 30-60 minutes. We will require the following:

- A short open-ended survey will be administered and used to collect biographical data at the onset of the study.
- A one-on-one online interview and will take approximately 30 to 60 minutes

For peer review only

Risks or Benefits

Direct benefits to you as an individual could include an opportunity to share your experiences and emotions during the COVID-19 pandemic. It is envisioned that the findings of this study could contribute towards an emergency care system to the advantage of the emergency care community.

Participation in the study could elicit negative emotions due to the sensitive nature of the topic and the emotional experiences you may have with it. In the event that you are experiencing discomfort as a result of any of the research processes, we will support you to obtain the psychological counselling / assistance you require. If you experience psychological discomfort during an interview, please bring this to the interviewer's attention and s/he will support you to obtain the assistance you require.

Confidentiality

All entries will be anonymised during the transcription, analysis and reporting processes. We will use a pseudonym to identify you. The researchers undertake to keep any information provided herein confidential, not to let it out of our possession and to report on the findings from the perspective of the participating group and not from the perspective of an individual.

Right to Refuse or Withdraw

You are not obliged to participate in this study if you do not want to do so. You may withdraw your participation at any time if you choose to do so, up until reporting of analyses. It is entirely your choice and no negative consequences will be incurred if you should choose not to participate or withdraw.

Future research

Anonymised transcribed data will be stored and will later be analysed to inform the initial phase of theory generation, thus contributing towards further related research. Such a theory can assist us in the future to understand the perceptions and experiences of frontline workers and to create systems that may better support them during pandemics.

Who to Contact

The research was reviewed and approved by the Human Research Ethics Committee of the University of Cape Town. The researcher team can be contacted during office hours at elzarie.theron@uct.ac.za. The UCT's Faculty of Health Sciences Human Research Ethics Committee can be contacted on 021 406 6338 in case you have any ethical concerns or questions about your rights or welfare as a participant on this research study.

For peer review only

PART 2: CONSENT

The perceptions and experiences of emergency medical staff on the impact of COVID-19 on trauma emergency care service provision in the Western Cape province of South Africa

I have read the foregoing information, or it has been read to me. I have had the opportunity to ask questions about it and any questions that I have had has been answered to my satisfaction. I consent voluntarily to participate as a participant in this interview. I understand that I may be identifiable.

The purpose of the recordings and the details of its storage and future analysis has been described to me. I have been offered to have any questions that I may have on the recording of the interview – answered and explained. I am giving consent voluntarily and I have been given a copy of this consent form.

Print Name of Participant: ______________________________

Signature of Participant: ______________________________

Date: ______________________________

A copy of this informed consent form has been provided to the participant.

Print Name of Researcher: ______________________________

Signature of Researcher: ______________________________

Date: ______________________________

For peer review only

APPENDIX 5: Consent Letter Study I

Lived experiences of emergency care personnel in the Western Cape, South Africa during the COVID-19 pandemic: a longitudinal hermeneutic phenomenological study

Investigators and affiliations

1. **Willem Stassen**, Division of Emergency Medicine, University of Cape Town
2. **Elzarie Theron**, Division of Emergency Medicine, University of Cape Town
3. **Helena Erasmus**, Department of Psychology, University of South Africa
4. **Craig Wylie**, Division of Emergency Medicine, Stellenbosch University
5. **Waseela Khan**, Division of Emergency Medicine, University of Cape Town
6. **Heike Geduld**, Division of Emergency Medicine, Stellenbosch University

You are invited to participate in a study conducted by the Division of Emergency Medicine at the University of Cape Town.

COVID-19 was declared a global pandemic in March 2020 following a cluster of pneumonia cases in Wuhan, China. Caused by severe acute respiratory syndrome Coronavirus -2 (SARS-CoV-2), COVID-19 continues to spread across the globe. The purpose of this study is to understand the lived experiences of emergency care personnel in the Western Cape. Understanding this perspective is necessary to mitigate adverse psychological and operational consequences of the COVID-19 pandemic by informing strategies to maintain appropriate emergency medical care through the life cycle of this pandemic, and in the planning for future pandemics. You were selected to participate in this study because you are working clinically on the frontline during the COVID-19 pandemic. Doctors, nurses and prehospital staff regardless of baseline qualification are eligible. We aim to recruit 12-16 frontline clinical providers in this study.

Voluntary Participation

Whether you decide to participate in the study or not is entirely your choice and voluntary. There will be no consequences to you if you decide not to participate. You may also decide to change your mind and may withdraw from participating, even if you agreed to it at an earlier stage.

Description of the Process

The study will take place over a period of three months and we will require your participation at various intervals during this period. If you choose to participate in this study, it will take up no more than 60 minutes of your time each week for the duration of the study. We will require the following:

- A short interviewer-administered open-ended survey will be used to collect biographical data at the onset of the study. The survey will be approximately 15 minutes long and will be conducted online, using an online communication tool for video calls.
- You will be asked to share their work schedule during the first interview and follow-up interviews and deadlines for data collection points will be planned accordingly.
- You will be asked to record a 15-30 minute voice recording reflecting on you lived experiences to date in the context of COVID-19. This should be submitted prior to their first pre-shift recording.

For peer review only

- You will be asked to record three to five-minute voice recordings on the following recommended times: 1) prior to a shift, 2) directly after a shift, and 3) 24 to 48 hours after the last shift in a cycle.
- One-on-one interviews will be conducted twice a month for the duration of the study. An online communications platform will be used to conduct the interviews, which will be approximately 40 to 60 minutes in length.
- You will be asked to complete the WHO (Five) Well-being scale every second week. The questionnaire will be available online and consists of a six-point Likert scale measuring positive and negative mental wellbeing.

Risks or Benefits

Direct benefits to you as an individual could include an opportunity to share your experiences and emotions during the COVID-19 pandemic. It is envisioned that the findings of this study will could contribute towards an emergency care system to the advantage of the emergency care community.

You will be reimbursed for the internet data that you use for the study, should you need it.

It is anticipated that the information we gain from this study will help us to reduce adverse psychological and operational consequences of the COVID-19 pandemic by informing strategies to maintain appropriate emergency medical care through the life cycle of this pandemic, and in the planning of future pandemics.

Participation in the study could elicit negative emotions or psychological responses. To monitor this and provide you with the assistance you need in such an event, you will be asked to end your reflections with an indication of how you are currently coping on a scale from 1 to 5 and your need for psychological assistance. In the event that you are experiencing discomfort as a result of any of the research processes, we will support you to obtain the psychological counselling / assistance you require. If you experience psychological discomfort during an interview, please bring this to the interviewer's attention and s/he will support you to obtain the assistance you require.

Confidentiality

In order to ensure that we can link subsequent entries and interviews to previously submitted entries, we need to link your identity. However, all entries will be anonymised during the transcription, analysis and reporting processes. We will use a pseudonym to identify you.

The researchers undertake to keep any information provided herein confidential, not to let it out of our possession and to report on the findings from the perspective of the participating group and not from the perspective of an individual.

Right to Refuse or Withdraw

You are not obliged to participate in this study if you do not want to do so. You may withdraw your participation at any time if you choose to do so, up until reporting of analyses. It is entirely your choice, and no negative consequences will be incurred if you should choose not to participate or withdraw.

Future research

For peer review only

Anonymised transcribed data will be stored and will later be analysed to inform the initial phase of theory generation, thus contributing towards further related research. Such a theory can assist us in the future to understand the experiences of frontline workers and to create systems that may better support them during pandemics.

Who to Contact

The research was reviewed and approved by the Human Research Ethics Committee of the University of Cape Town. The researcher team can be contacted during office hours at elzarie.theron@uct.ac.za. Should you have any questions regarding the ethical aspects of the study, you can contact the Human Research Ethics Committee of the University of Cape Town, South Africa; contact Tel: +27 21 650 3002, Email: hrec-enquiries@uct.ac.za.

Part 2: Consent

Lived experiences of emergency care personnel in the Western Cape, South Africa during the COVID-19 pandemic: a longitudinal hermeneutic phenomenological study

I have read the foregoing information, or it has been read to me. I have had the opportunity to ask questions about it and any questions that I have had has been answered to my satisfaction. I consent voluntarily to participate as a participant in this interview. I understand that I may be identifiable.

The purpose of the recordings and the details of its storage and future analysis has been described to me. I have been offered to have any questions that I may have on the recording of the interview – answered and explained. I am giving consent voluntarily and I have been given a copy of this consent form.

Print Name of Participant: ____
Signature of Participant: ____
Date: ____

A copy of this informed consent form has been provided to the participant.

Print Name of Researcher: ____
Signature of Researcher: ____
Date: ____

For peer review only

Standards for Reporting Qualitative Research (SRQR)*

http://www.equator-network.org/reporting-guidelines/srqr/

Title and abstract

**Title** - Concise description of the nature and topic of the study Identifying the study as qualitative or indicating the approach (e.g., ethnography, grounded theory) or data collection methods (e.g., interview, focus group) is recommended	Page 1
**Abstract** - Summary of key elements of the study using the abstract format of the intended publication; typically includes background, purpose, methods, results, and conclusions	Page 2

Introduction

**Problem formulation** - Description and significance of the problem/phenomenon studied; review of relevant theory and empirical work; problem statement	Page 4 and 5
**Purpose or research questio**n - Purpose of the study and specific objectives or questions	Page 6

Methods

**Qualitative approach and research paradigm** - Qualitative approach (e.g., ethnography, grounded theory, case study, phenomenology, narrative research) and guiding theory if appropriate; identifying the research paradigm (e.g., postpositivist, constructivist/ interpretivist) is also recommended; rationale**	Page 7
**Researcher characteristics and reflexivity** - Researchers' characteristics that may influence the research, including personal attributes, qualifications/experience, relationship with participants, assumptions, and/or presuppositions; potential or actual interaction between researchers' characteristics and the research questions, approach, methods, results, and/or transferability	Page 10
**Context** - Setting/site and salient contextual factors; rationale**	Page 6
**Sampling strategy** - How and why research participants, documents, or events were selected; criteria for deciding when no further sampling was necessary (e.g., sampling saturation); rationale**	Page 7
**Ethical issues pertaining to human subjects** - Documentation of approval by an appropriate ethics review board and participant consent, or explanation for lack thereof; other confidentiality and data security issues	Page 8
**Data collection methods** - Types of data collected; details of data collection procedures including (as appropriate) start and stop dates of data collection and analysis, iterative process, triangulation of sources/methods, and modification of procedures in response to evolving study findings; rationale**	Page 8

For peer review only - http://bmjopen.bmj.com/site/about/guidelines.xhtml

**Data collection instruments and technologies** - Description of instruments (e.g., interview guides, questionnaires) and devices (e.g., audio recorders) used for data collection; if/how the instrument(s) changed over the course of the study	Page 8
**Units of study** - Number and relevant characteristics of participants, documents, or events included in the study; level of participation (could be reported in results)	Page 7 and 8
**Data processing** - Methods for processing data prior to and during analysis, including transcription, data entry, data management and security, verification of data integrity, data coding, and anonymization/de-identification of excerpts	Page 9
**Data analysis** - Process by which inferences, themes, etc., were identified and developed, including the researchers involved in data analysis; usually references a specific paradigm or approach; rationale**	Page 9 and 10
**Techniques to enhance trustworthiness** - Techniques to enhance trustworthiness and credibility of data analysis (e.g., member checking, audit trail, triangulation); rationale**	Page 10

Results/findings

**Synthesis and interpretation** - Main findings (e.g., interpretations, inferences, and themes); might include development of a theory or model, or integration with prior research or theory	Page 10-15
**Links to empirical data** - Evidence (e.g., quotes, field notes, text excerpts, photographs) to substantiate analytic findings	Page 10-15

Discussion

**Integration with prior work, implications, transferability, and contribution(s) to the field** - Short summary of main findings; explanation of how findings and conclusions connect to, support, elaborate on, or challenge conclusions of earlier scholarship; discussion of scope of application/generalizability; identification of unique contribution(s) to scholarship in a discipline or field	Page 15-18
**Limitations** - Trustworthiness and limitations of findings	Page 18-19

Other

**Conflicts of interest** - Potential sources of influence or perceived influence on study conduct and conclusions; how these were managed	Page 20
**Funding** - Sources of funding and other support; role of funders in data collection, interpretation, and reporting	Page 20

*The authors created the SRQR by searching the literature to identify guidelines, reporting standards, and critical appraisal criteria for qualitative research; reviewing the reference lists of retrieved sources; and contacting experts to gain feedback. The SRQR aims to improve the transparency of all aspects of qualitative research by providing clear standards for reporting qualitative research.

For peer review only

**The rationale should briefly discuss the justification for choosing that theory, approach, method, or technique rather than other options available, the assumptions and limitations implicit in those choices, and how those choices influence study conclusions and transferability. As appropriate, the rationale for several items might be discussed together.

Reference:
O'Brien BC, Harris IB, Beckman TJ, Reed DA, Cook DA. **Standards for reporting qualitative research: a synthesis of recommendations.** *Academic Medicine*, Vol. 89, No. 9 / Sept 2014
DOI: 10.1097/ACM.0000000000000388

For peer review only

BMJ Open

Companion Restrictions in the Emergency Department during COVID-19: Physician Perceptions from the Western Cape, South Africa

Journal:	*BMJ Open*
Manuscript ID	bmjopen-2022-070982.R1
Article Type:	Original research
Date Submitted by the Author:	24-Mar-2023
Complete List of Authors:	Wiebe, Lauren; Karolinska Institutet, Department of Global Public Health<br>Alvesson, Helle; Karolinska Institutet, Department of Global Public Health<br>Stassen, Willem; University of Cape Town, Division of Emergency Medicine
**Primary Subject Heading**:	Qualitative research
Secondary Subject Heading:	Infectious diseases, Emergency medicine, Global health
Keywords:	COVID-19, ACCIDENT & EMERGENCY MEDICINE, Protocols & guidelines < HEALTH SERVICES ADMINISTRATION & MANAGEMENT, QUALITATIVE RESEARCH, Clinical Decision-Making, Physicians

SCHOLARONE™ Manuscripts

For peer review only - http://bmjopen.bmj.com/site/about/guidelines.xhtml

I, the Submitting Author has the right to grant and does grant on behalf of all authors of the Work (as defined in the below author licence), an exclusive licence and/or a non-exclusive licence for contributions from authors who are: i) UK Crown employees; ii) where BMJ has agreed a CC-BY licence shall apply, and/or iii) in accordance with the terms applicable for US Federal Government officers or employees acting as part of their official duties; on a worldwide, perpetual, irrevocable, royalty-free basis to BMJ Publishing Group Ltd ("BMJ") its licensees and where the relevant Journal is co-owned by BMJ to the co-owners of the Journal, to publish the Work in this journal and any other BMJ products and to exploit all rights, as set out in our licence.

The Submitting Author accepts and understands that any supply made under these terms is made by BMJ to the Submitting Author unless you are acting as an employee on behalf of your employer or a postgraduate student of an affiliated institution which is paying any applicable article publishing charge ("APC") for Open Access articles. Where the Submitting Author wishes to make the Work available on an Open Access basis (and intends to pay the relevant APC), the terms of reuse of such Open Access shall be governed by a Creative Commons licence – details of these licences and which Creative Commons licence will apply to this Work are set out in our licence referred to above.

Other than as permitted in any relevant BMJ Author's Self Archiving Policies, I confirm this Work has not been accepted for publication elsewhere, is not being considered for publication elsewhere and does not duplicate material already published. I confirm all authors consent to publication of this Work and authorise the granting of this licence.

For peer review only

Companion Restrictions in the Emergency Department during COVID-19: Physician Perceptions from the Western Cape, South Africa

For peer review only

Lauren E Wiebe[1], Helle Mölsted Alvesson[1], Willem Stassen[2]

1 Department of Global Public Health, Karolinska Institutet, Stockholm, Sweden

For peer review only - http://bmjopen.bmj.com/site/about/guidelines.xhtml

2 Division of Emergency Medicine, University of Cape Town, Cape Town, South Africa

Corresponding author:

Helle Mölsted Alvesson

Karolinska Institutet, SE-171 77 Stockholm, Sweden

helle.molsted-alvesson@ki.se

Word Count: 4,300

For peer review only

ABSTRACT

Objectives: To determine emergency department physicians' perceptions regarding hospital companions being prohibited from accompanying the patient during COVID-19.

Design: Two qualitative datasets were combined. Data collected included voice recordings, narrative interviewing, and semi-structured interviews. A reflexive thematic analysis was conducted and guided by the Normalisation Process Theory.

Setting: Six hospital Emergency Departments in the Western Cape, South Africa.

Participants: Convenience sampling was used to recruit a total of eight physicians working full-time in the emergency department during COVID-19.

Results: The lack of physical companions provided an opportunity for physicians to assess and reflect on a companion's role in efficient patient care. Physicians perceived that the COVID-19 restrictions illuminated that patient companions engaged in the emergency department as providers contributing to patient care by providing collateral information and patient support, while simultaneously engaging as consumers detracting physicians from their priorities, and patient care. These restrictions prompted the physicians to consider how they understand their

For peer review only

patients largely through the companions. When companions became virtual, the physicians were forced to shift how they perceive their patient, which included increased empathy.

Conclusion: The reflections of providers can feed into discussions about values within the health care system and can help explore the balance between medical and social safety, especially with companion restrictions still being practiced in some hospitals. These perceptions illuminate various tradeoffs physicians had to consider throughout the pandemic and may be used to improve companion policies when planning for the continuation of the COVID-19 pandemic and future disease outbreaks.

For peer review only

STRENGTHS AND LIMITATIONS OF THIS STUDY

- COVID-19 caused this study to pivot from purposive sampling to convenience sampling, potentially introducing a sampling bias affecting transferability and trustworthiness of the results.
- One professional base was included, potentially introducing a physician bias which can limit the transferability of this study.
- In-depth knowledge of one professional base was sampled at different time points between August 2020 to January 2022 providing a sufficient period to capture changed practices.
- By combining and comparing two datasets with different types of data, we were able to capture a wider range of perceptions, reinforcing our situational and reflexive data. This has strengthened the quality and dependability of our analysis and results in relation to our research aim.

For peer review only

INTRODUCTION

Emergent conditions represent approximately 90% of global mortality (1). High functioning emergency departments (ED) are vital to alleviating this burden, but their operations are influenced by the greater environment, social context, and disease burden (1–3).

A variety of factors contribute to successful emergency departments. The World Health Organization (WHO) provides Essential Trauma Care Guidelines and emphasizes the importance of the implementation of standard policies and protocols in acute care facilities (4). Policies and protocols take time to establish, and the tumultuous nature of COVID-19 posed challenges for maintaining and adapting standardised procedures (5). Additionally, EDs often face challenges with communication and mismanaged information. Health professionals in EDs spend 80% of their time engaging with communication processes, including with colleagues, management, patients, and companions (6). In a high-risk, fast-paced environment like EDs, having

For peer review only

established communication and information pathways is critical to patient care; given that up to 12% of errors in EDs are attributed to inadequate communication (6–8).

Prior to COVID-19, patient companions were prominent participants of communication in EDs. The literature highlights the strengths and challenges associated with this (9–13). Studies show companions interfering with patient care by causing interruptions for the medical team, potentially leading to clinical errors, as well as increasing the stress on health care professionals when performing life-saving procedures (9,10). However, communication with companions is vital for gaining a quick medical history to inform effective care (9,10). Furthermore, the companion is a key component of support for both the patient and family, resulting in greater patient motivation for recovery, reduced length of stay and less fear and anxiety for both the patient and family (9,11–13). The literature published prior to COVID-19 showed mixed findings on health professionals' perspective of patient companions. Many studies found nurses more in favour of familial presence than physicians, especially in emergency situations such as resuscitation (10,12).

During COVID-19, to reduce transmission rates, countries globally implemented policies that limited, if not completely restricted companions or escorts accompanying hospitalized

For peer review only

patients, which resulted in both positive and negative outcomes (14,15). Studies have shown various impacts on the patient such as mental health challenges as well as reduced hygiene and nutritional status (14–16). Companions and escorts also experienced many emotional and mental consequences including depression, fear, and anger (15). Lastly, providers were impacted by the increasing demands for communication from families, and the stress of facilitating virtual communication (15,17). However, studies also reported positive impacts including less crowding, increased space for social distancing and reduced transmission amongst patients, companions, and providers (18,19). Overall, most existing studies do not support universal restrictive policies in clinical settings during COVID-19, on the basis that the risks outweigh the benefits. The primary risks identified include psychological deficits to the patient and family, worsened patient health outcomes, distress in connection with end-of-life care and increased stress for providers (14–16,20). Additionally, the evidence of reduced transmission rates is limited in quality (14–16,20).

As of June 2022, South Africa has had approximately 4 million cases and 102,000 COVID-19 related deaths (21). The impacts of COVID-19 in South Africa, specifically the Western Cape, are vast, including increased mortality of COVID-19 and other diseases, delayed

For peer review only

health-seeking behaviour, and changes in injury presentation (22). EDs are uniquely impacted by COVID-19 and companion restrictions because they frequently serve already vulnerable populations (5). Additionally, emergent conditions are dynamic in nature making it harder to consistently implement new and evolving COVID-19 regulations (5).

Although there is existing research on the broad COVID-19 impacts, the effects of companion restrictions throughout the COVID-19 pandemic have been minimally explored, especially outside pediatric and palliative settings (15,16,19). In addition, the perspective of emergency health providers is rarely included. The study aim was to determine emergency department physicians' perceptions regarding hospital companions and escorts being prohibited from accompanying the patient during COVID-19.

METHODS

South Africa is known for having some of the highest numbers of trauma in the world, in addition to experiencing a quadruple burden of disease (1,23,24). This study is specifically taking place in the Western Cape, South Africa because a high proportion of the countries' emergent conditions and diseases present in EDs here (23). It was also the first province within South Africa to experience a COVID-19 surge and has maintained a relatively high rate of cases

For peer review only

and deaths, with approximately 21% of cases and 22% of COVID-19 related deaths in the country (25). COVID-19 affected practices that contribute to successful care in South African EDs, such as companion restrictions, structurally splitting the ED into COVID-19 likely and COVID-19 unlikely, reduction of blood supply, shortage of PPE and the redistribution of health professionals leaving many departments with insufficient human resources (26,27). The high number of emergent conditions and excessive burden of COVID-19 made it a unique and appropriate setting to evaluate the impacts of COVID-19.

Study Design

The study utilised two different qualitative datasets. Dataset I included data from the end of the first wave of COVID-19 in August to October 2020 and explored emergency medical personnel's lived experiences with the pandemic (28). Dataset II was collected during the second and third waves, from February 2021 to January 2022, and included semi-structured interviews of physicians' perceptions on how COVID-19 affected emergency care.

Sampling and Recruitment

Both datasets included in-hospital emergency personnel in the Western Cape. Convenience sampling was utilised, and emergency medical joint staff were contacted

For peer review only

using their registered email addresses in the UCT staff repository. Staff were asked to distribute the study invitation to their employees for equal access. Subsequent participants were collected through snowball sampling. Our proposed sample size was 12 participants, but our priority was reaching information saturation, which was assessed throughout the data collection process. Our final sample included eight participants, who worked full-time in the emergency department throughout COVID-19, from six different hospitals.

The mean age of participants was 35 years old, with 9 years of medical experience. Demographic information was gained through a verbal questionnaire. The sample characteristics are included in table 1 while maintaining anonymity (Table 1).

Table 1 - Participant ID Codes

ID Code	Self-Identified Gender	Job Title	Years in profession (since qualification)
ID1	Male	Medical officer	9
ID2	Female	Consultant	11
ID3	Female	Consultant	5
ID4	Female	Medical officer	11
ID5	Female	Registrar	6
ID6	Male	Medical officer	8
ID7	Male	Registrar	9
ID8	Female	Registrar	8

For peer review only

Data Collection

This study aimed to answer two questions: (1) Which practices in the emergency department do the physicians perceive as being changed by the restrictions on companions/escorts throughout COVID-19 and (2) how have the restrictions impacted how physicians conceptualize the role of companions in patient care? Dataset I included WhatsApp voice recordings and one-on-one online interviews, conducted in English or Afrikaans. Only physicians from this dataset were included. Voice recordings ranged from 17 seconds to 30 minutes, and interviews ranged from 40-60 minutes. In dataset II, a semi-structured interview guide (Appendix 1) was used in all interviews and was inspired by the Normalisation Process Theory (NPT). The interview guide was piloted for clarity with an emergency physician in Sweden and was adjusted accordingly. The interviews were conducted online in English and ranged from 40-60 minutes.

Ethical approval was obtained from the University of Cape Town's Faculty of Health Sciences Human Research Ethics Committee (HREC ref. 052/2021) (Appendix 2). The ethical application for dataset I (HREC ref. 386/2020) included approval for the anonymised data to be reanalysed for similar research questions (Appendix 3). All participants were provided with an

For peer review only

information and consent letter, and they were able to ask any questions before signing (Appendix 4,5). Further approval was sought from the Provincial Department of Health and the hospitals for participants to feel safe participating, with no negative repercussions in their workplace.

Patient and Public Involvement

We had no patient/public involvement.

Analysis

The study aim and questions were inspired by Reflexive Monitoring which has been identified as an important feature in qualitative improvements, as it highlights how people assess the impacts of new practices on themselves and others (29). In our context, we are examining how physicians assess the impacts of companion restrictions in their personal practice, along with the appraisal of their colleagues, companions, and the patient. Reflexive Monitoring is a part of the Normalisation Process Theory (NPT), which focuses on the processes behind changes in thinking, practices, policies and organisation in healthcare settings, specifically resource-constrained contexts (29).

(Figure 1).

For peer review only

The data from both studies were subjected to an inductive, reflexive thematic analysis using the six steps as described by Braun and Clarke (30). NVivo V.12 Pro (QSR International Pty Ltd, Australia) was used to develop the main categories and themes. Transcripts were initially coded by one data collector and a list of latent codes was developed. After the interviews were coded, subcategories were discussed in relation to emerging themes and were narrowed down to identify the main theme(s). These steps were done as an iterative process, where the codes and emerging themes were continuously reviewed amongst the research team, to help achieve triangulation and comprehensive analysis (31). Throughout the analysis, the data was reviewed in relation to reflexive monitoring in the NPT. However, this was used to reinforce, not determine, or limit the themes. Anonymity was prioritized in this study and each participant was given an ID code to ensure their information was not linked to their responses or quotations. Pseudonyms were used in the transcriptions.

Reflexivity

The research group was made up of an interdisciplinary team, all with experience with qualitative studies in Cape Town. In addition, one of our primary investigators has extensive

For peer review only

research experience within emergency medicine. In qualitative studies, the interviewer's position and relationship with participants can impact the comfort and openness of participants (32). The interviewer was a master's student of public health, with no ties to the hospitals that the physicians worked at. This potentially contributed to building trust and the physicians feeling more comfortable to share information about their workplace.

RESULTS

During COVID-19, the restriction was implemented that no companion was allowed to accompany the patient upon arrival to the ED to reduce the risk of COVID-19 transmission (27). In the absence of physical companions, practices surrounding patient support, physician and patient communication with companions and collection of collateral information were disrupted as companions became virtual. Based on physician perceptions, this change illuminated the different roles physical companions hold in patient care as both providers contributing to patient care, and consumers of care, potentially detracting attention from the patient in the ED (Table 2).

Table 2 – Results Table

For peer review only

Theme	Reconceptualising Companions in the Emergency Department		
Category	Virtual Companions		Finding Balance
	Companions as Providers	Companions as Consumers	
Codes	Patient support<br>Collateral information<br>Nature of communication<br>Provider empathy	Interruptions<br>Crowding<br>Call volume<br>Time consumption	Visiting hours<br>Technology<br>Contact sheets

Companions as Providers in the ED

Pre-COVID, physicians relied on patients being physically accompanied to the ED to be able to communicate with companions. Physicians acknowledged that companions play an important role for both the patient and the provider in the ED. When companions were not physically present, it often resulted in the patient being dependent on the health provider to communicate with the family on their behalf.

"A lot of them [patients] would not necessarily have a cell phone or a charged cell phone, or data to contact the families. And so, there were a lot of them that were very anxious and that definitely made it difficult for us."- ID1

The physicians expressed that the prominent negative aspect of not having physical companions were the challenges associated with obtaining collateral patient

For peer review only

information relevant for clinical care, including pre-existing conditions, family history and medications. They viewed this as problematic to their ability to provide care.

"Having a physical escort there is often the only way to get collateral about a patient, and especially during COVID, so much about your decision about the level of care, and level of intervention was based on a patient's baseline … when these people didn't answer their phones, or you didn't have those numbers, it was literally impossible to make these kinds of decisions." – ID5

Physicians expressed that getting a hold of the companions was often time-consuming and occasionally, they could not get a hold of them at all, meaning they were having to make decisions regarding care without that critical information. Physicians also reflected on companion presence pre-COVID and acknowledged that the physical presence resulted in higher quality and more individualized care planning, compared to trying to get a hold of companions virtually.

"This meant a lot more digging in folders looking for ID numbers, trying to contact family members outside of the hospital, phones that weren't answered, that really made an impact on how we manage patients and it also delayed turnaround time. Which meant we had patients boarding a lot longer in an emergency unit."- ID6

Companions as Consumers in the ED

For peer review only

Although physicians acknowledged various ways that companions as consumers contribute to and enhance practices in the ED, the shift to virtual companions also illuminated ways that physical companions interfered with different practices. One reason for why physicians expressed a preference for the virtual companionship introduced during COVID-19 was that they viewed patient treatment as their primary task and having virtual companions increased the efficiency of their clinical tasks. Specifically, due to fewer interruptions and less crowding while treating the patient, they felt they were able to prioritize patient care, resulting in fewer mistakes.

"Before there were two visiting times per day, an hour-long visiting time, which means that every patient in the unit could have two people within that hour, which literally triples the amount of people in the room. So, it's also just getting in the way, it's two hours of the day where you can't do anything because there's no point in being there." – ID5

"So not having those people to kind of judge you for what was going on definitely took off the stress of the work to some degree, because you could kind of get on with what you needed to do." - ID8

To compensate for the lack of physical presence, EDs implemented various practices such as contact sheets upon arrival, tick sheets, involving other departments such as psychology or having a dedicated health provider conducting all the calls to

For peer review only - http://bmjopen.bmj.com/site/about/guidelines.xhtml

families. Physicians described being able to communicate suggestions for different solutions to the consultant level, who worked with management to implement different systems. However, physicians felt these methods frequently resulted in calls not being conducted, or the physician completing the call themselves. It also created increased unrest amongst the virtual companions, resulting in a higher number of calls to the ED overwhelming their phone system, a phone system designated for emergent referrals. The in-person interruptions that occurred pre-COVID, shifted to the phone, especially with many companions wanting to speak to the physician directly, and not other providers. The time-consuming nature of these changes resulted in the physician having less time for tasks related to patient care.

"The phone calls to the hospital were very difficult to handle because sometimes it'd be 22 family members of the same patient who all wanted a detailed update from the doctor. Each of which identified themselves as the family spokesperson." – ID3

Separate from the frequency of communication, physicians described the nature of communication with companions shifting throughout COVID-19 due to the severity and rapid changes in the patient's condition. Physicians acknowledged that dealing with companions virtually rendered these conversations more difficult. In addition to

For peer review only

changing the nature of their communication with companions, it impacted how the provider interacted with the patient, including being more selective with their words and increasing compassion and empathy.

"It's probably one of the hardest things I've ever had to do to phone so many families and tell them that their family members died or was going to die or wasn't going to make it…I think I definitely learned to kind of find the compassion even when you're tired and exhausted." - ID8

Finding Balance for Companion Engagement

Beyond the time-consuming nature of updating virtual companions, physicians were able to identify both benefits and challenges associated with virtual companionship, throughout COVID-19 and within the context of the ED in general. They expressed that finding a hybrid of pre-COVID practices and COVID-19 restrictions moving forward is necessary for providers, patients, and companions. Physicians offered up different suggestions such as increasing the capacity to facilitate video calling, limiting the number of companions at the bedside, allowing one designated patient spokesperson at the bedside, reducing the length of visiting hours and having companions fill in up to date contact information when dropping off patients.

"We need to find something that works a bit better for families, but also allows us to actually see our patients timely and not have patients waiting for 12 hours to see a doctor, those kinds of things." – ID3

"We sometimes easily lack the human element in the emergency department when pressed for beds and patients and just chaos that is generally in the ED… There was across the board, from all my colleagues, a much more human aspect to that kind of [patient] interaction, than there were before."-1D7

Physicians mentioned that COVID-19 changes, specifically patients having no companions, illuminated inadequate practices in EDs that must balance humanity factors with the dynamic nature of emergency medicine. Physicians acknowledged finding a way for these factors to co-exist is an important lesson to bring forward throughout and post-COVID-19.

DISCUSSION

COVID-19 caused many formal and informal changes that providers had to juggle in conjunction with companion restrictions. Formal changes included structurally splitting the ED, different clinical protocols and prohibiting patient companions. Informal changes included disrupting tacit team knowledge, provider decision-making and communication adaptations. Physicians provided insight from their perspective on the impacts companion restrictions had on the ED, which can help inform future change

For peer review only

and preparedness. Physicians were faced with a new scenario of no patient companions, yielding a paradox as the efficiency and quality of patient care simultaneously improved and suffered with virtual companions engaging as both providers and consumers in the ED. Emergency physicians' perceptions of the lacking physical presence of companions emerges as an understudied area, specifically in Eds, and should be incorporated into the greater debate on patient companions. Existing literature primarily focuses on companions from a patient-centered care perspective, as companions contribute to feelings of patient agency, safety, and improved health outcomes (11–13). Totten et al., (2014) found that 78% of patients in the ED felt their companion was important to their care for social support and advocacy (13). Although there are no official guidelines for companions in the ED, there is a large body of literature in support of companion presence in other health care settings such as maternal care and the ICU (33,34). For example, the WHO has issued recommendations for companions of choice to be present during labour and childbirth and argues that having a companion they trust contributes to women feeling safe and strong and leads to improved maternal and perinatal outcomes.

For peer review only

One of the main factors they attribute this to is that companions bridge communication between the healthcare professionals and women (34).

Overall, physicians acknowledged both benefits and challenges with virtual companions, and expressed the desire, in moving forward, to find a balance between the policies and practices pre-COVID and during COVID-19. Iness et al. and Herbst et al. conducted studies that include the physician perspective in the ICU during COVID-19 and found that physicians perceived virtual companions and telecommunication to increase their workload and limit effective and empathetic communication (20,35). This aligns with our findings that physicians noticed virtual companions to be more time-consuming. However, there are few studies that include the physician perspective, and none conducted specifically in the ED. Therefore, more research is needed to situate our specific findings, in order to propose comprehensive and sustainable solutions moving forward.

In addition to companion restrictions, physicians explained that COVID-19 patients were not moved to isolation facilities, but instead separated within the hospital into “COVID likely” and “COVID unlikely” areas in EDs based on their respiratory

For peer review only

symptoms. The literature from other recent disease outbreaks such as SARS and Ebola, predominantly supports physical companion presence on the basis that it yields greater benefits to the patient than risks (36). Itis argued that restricting companions can discourage patients from seeking care, which can undermine infection control efforts, as found in the Ebola response (37). When positioning our results within the literature on companion restrictions during disease outbreaks, our findings challenge whether hospitals and health systems have sufficiently applied lessons learned about companions from previous outbreaks. There needs to be increased incorporation of companion restriction research for future emergency preparedness and response planning.

Participating physicians perceived their primary role to be healthcare providers, treating the patient and thought that having virtual companions was beneficial as it reduced interruptions, errors, and stress. Results are quite divided from the limited ED literature on health providers' views of companions under normal circumstances. Timmermans and Meyers et al., propose that health professionals perceive companions as a hindrance to the process of saving a patient's life (38). Generally, physicians were more worried than other health professions about the presence of companions in terms of

For peer review only

crowding and interruptions. According to Readly and Hood, 61% of health professionals felt they would have increased anxiety and stress in life-saving procedures with companions' present (38). The alignment of our findings with the literature illuminates the importance of including physician input when implementing policies, as they impact physician performance and subsequently patient care and safety. Further research needs to be conducted to find a balance which respects the physician's perspective, while also protecting patient-centred care.

Physicians perceived companions to be important for gaining collateral information about the patient. In the ED focused literature, physicians primarily did not perceive companions to be associated with patient care, even though they too understand them to provide critical health information as the patient's spokesperson (38). However, there is a knowledge gap regarding the patient care effects and potential added inefficiency associated with physical companions being switched to virtual communication. This gap could be attributed to the fact that most existing literature does not concern provider populations that have experienced companion restrictions before.

For peer review only

Therefore, they have never actively had to consider how care and efficiency would shift when the companions switch to being virtual.

COVID-19 provided an opportunity for physicians to engage with reflexive monitoring as they appraise the role of companions, having had a prolonged experience without them. Moving forward, there is an opportunity for further research into whether these experiences have led to changes in how physicians perceive companions as an important component of care.

Strengths and Limitations

This study obtained in-depth knowledge of one professional base sampled at different time points from August 2020 to January 2022, which provides a sufficient period to capture changed practices.

This study faced challenges with recruitment attributed to the delays caused by the COVID-19 vaccine roll-out, which put research project approvals on hold. Due to the extenuating circumstances, this study had to pivot from purposive to convenience sampling, which can introduce a sampling bias caused by lack of equal access to participation, thereby affecting the transferability and trustworthiness of the results (39).

For peer review only

Additionally, these circumstances resulted in a smaller sample size, potentially introducing selection bias. However, to minimize the impacts, we chose to maximize saturation by focusing on the perceptions of one professional base. Our sample included experienced physicians, from multiple hospitals, who were able to compare practices before and during the pandemic. However, since COVID-19 is ongoing, we are not able to exhaustively capture how the physicians continue to perceive the changes. Additionally, we acknowledge the physician bias limits the transferability of this study.

This study combined two qualitative datasets that utilised different interview methodologies. This may have posed challenges for analysis because it introduced the potential for bias when coding dataset I. It did not involve the use an interview guide, since the coder may then have been inclined to look for codes or themes that align with the main topics from the dataset II interview guide. This bias was reduced by continuously discussing the data amongst the research team. However, both studies focused on gathering perceptions from in-hospital emergency physicians. By combining these two datasets, with different types of data, we were able to capture a wider range of perceptions. In the voice recordings, physicians were able to share their perceptions in

For peer review only

real-time pre and post shift, allowing relevant situational information to be shared, that might have been forgotten by the time of a follow-up interview. We were able to compare both datasets, reinforcing our situational and reflexive data which has strengthened the quality and dependability of our analysis and results in relation to our research aim (31).

Implications of the Study

Learning from physicians' experiences and perspectives can help create a more holistic understanding of the contribution of the companion in the ED. This can inform companion policies that will promote preparedness, patient-centred care, and improved health outcomes in the everyday context, in preparing for the continuation of the COVID-19 pandemic and in future disease outbreaks. It also provides a platform for future research to increase emergency preparedness. Specifically, to capture more comprehensive results, the study can be continued with purposive sampling, increased sample size and the inclusion of more professional bases, such as nurses, to gain a wider perspective of the companion restrictions. In addition, follow up studies can be conducted once COVID-19 has settled to learn how these changes were normalised.

For peer review only

CONCLUSION

Physicians were introduced to the benefits and challenges associated with virtual companions. They felt the benefits of reduced crowding and interruption of bedside care and the challenges associated with the lack of collateral information and the time-consuming nature of virtual communication with companions. Overall, physicians expressed their hope that policies achieving a hybrid of pre-COVID and COVID practices will be sustained moving forward, including the use of contact information sheets, limitation of the number of bedside companions, and increased access to video technology. By learning from their perceptions, there is an opportunity to better prepare the ED and providers for the next pandemic or other crisis that may affect companion policies, to maintain the highest level of patient centered care.

For peer review only

Acknowledgements

The authors wish to thank all the physicians who participated and shared their experiences. We would like to acknowledge Elzarie Theron and Helena Erasmus for their contribution to the data collection from dataset I.

Figure Legend

Figure 1.1 – visual representation of the Normalisation Process Theory.

Contributorship Statement

All authors contributed to the conception and design of the study. LW was the primary contributor to data collection, data analysis and interpretation, paper writing, revisions, and submission. HMA contributed to the qualitative methodology design, data analysis and interpretation, as well as draft, and final submission revisions. WS contributed to sampling and recruitment, draft and final paper revisions, data sharing and providing the final approval for submission. All authors agree to be accountable for their work.

Competing Interests

The authors have declared that no conflict of interests exist.

Funding

None to report.

Data Sharing Agreement

Within the transcripts, physicians discuss personal and potentially identifying experiences from their workplace. The informed consent that all physicians signed promised full anonymity. Therefore, making the full data set publicly available would potentially breach their privacy. Following data requests, transcripts will be reviewed for any potential identifying information and will only be made available to researchers who sign a data sharing agreement. Data requests may be sent to Willem Stassen at willem.stassen@uct.ac.za.

Ethics Approval Statement

This study involves human participants and was approved by University of Cape Town's Faculty of Health Sciences Human Research Ethics Committee (HREC ref. 052/2021).

References

1. Chang CY, Abujaber S, Reynolds TA, Camargo CA, Obermeyer Z. Burden of emergency conditions and emergency care usage: New estimates from 40 countries. Emergency Medicine Journal. 2016 Nov 1;33(11):794–800.

For peer review only

2. Gunning AC, Lansink KWW, Van Wessem KJP, Balogh ZJ, Rivara FP, Maier R V., et al. Demographic Patterns and Outcomes of Patients in Level i Trauma Centers in Three International Trauma Systems. World J Surg. 2015 Nov 1;39(11):2677–84.

3. Reynolds TA, Stewart B, Drewett I, Salerno S, Sawe HR, Toroyan T, et al. The Impact of Trauma Care Systems in Low-and Middle-Income Countries. Annu Rev Public Health. 2017;38:507–39.

4. Word Health Organization. Guidelines for Essential Trauma Care. 2004.

5. Haut ER, Leeds IL, Livingston DH. The Effect on Trauma Care Secondary to the COVID-19 Pandemic. Ann Surg. 2020 Sep;272(3):e204–7.

6. Creswick N, Westbrook JI, Braithwaite J. Understanding communication networks in the emergency department. BMC Health Serv Res. 2009 Dec 31;9(1):247.

7. Pun JKH, Matthiessen CMIM, Murray KA, Slade D. Factors affecting communication in emergency departments: doctors and nurses' perceptions of communication in a trilingual ED in Hong Kong. Int J Emerg Med. 2015 Dec 1;8(1):1–12.

8. Reddy MC, Paul SA, Abraham J, Mcneese M, Deflitch C, Yen J. Challenges to effective crisis management: Using information and communication technologies to coordinate emergency medical services and emergency department teams. 2008;

9. Kilner E, Sheppard LA. The role of teamwork and communication in the emergency department: A systematic review. Int Emerg Nurs. 2010 Jul 1;18(3):127–37.

10. Porter J, Cooper SJ, Sellick K. Attitudes, implementation, and practice of family presence during resuscitation (FPDR): A quantitative literature review. Int Emerg Nurs. 2013 Jan 1;21(1):26–34.

11. Berwick D, Downey A, Cornett E, Sector C on MTCLHS and IT to the C, Policy B on HS, Populations B on the H of S, et al. Delivering Patient-Centered Trauma Care. 2016 Sep 12;

12. Olding M, McMillan SE, Reeves S, Schmitt MH, Puntillo K, Kitto S. Patient and family involvement in adult critical and intensive care settings: a scoping review. Health Expectations. 2016 Dec 7;19(6):1183–202.

13. Totten VY, Bryant TK, Chandar AK, Hoch WB, Hunter SL, Patel NJ, et al. Perspectives on Visitors in the Emergency Department: Their Role and Importance. J Emerg Med. 2014 Jan 1;46(1):113–9.

For peer review only

14. Jaswaney R, Davis A, Cadigan RJ, Waltz M, Brassfield ER, Forcier B, et al. Hospital Policies during COVID-19: An Analysis of Visitor Restrictions. Journal of Public Health Management and Practice. 2021;

15. Hugelius K, Harada N, Marutani M. Consequences of visiting restrictions during the COVID-19 pandemic: An integrative review. Int J Nurs Stud [Internet]. 2021 Sep 1 [cited 2022 Apr 24];121:104000. Available from: /pmc/articles/PMC8196532/

16. Lo AX, Wedel LK, Liu SW, Wongtangman T, Thatphet P, Santangelo I, et al. COVID-19 hospital and emergency department visitor policies in the United States: Impact on persons with cognitive or physical impairment or receiving end-of-life care. J Am Coll Emerg Physicians Open [Internet]. 2022 Feb [cited 2022 Apr 24];3(1). Available from: /pmc/articles/PMC8776041/

17. Fino E, Fino V, Bonfrate I, Russo PM, Mazzetti M. Helping patients connect remotely with their loved ones modulates distress in healthcare workers: a tend-and-befriend hypothesis for COVID-19 front liners. Eur J Psychotraumatol. 2021;12(1).

18. Wendlandt B, Kime M, Carson S. The impact of family visitor restrictions on healthcare workers in the ICU during the COVID-19 pandemic. Intensive Crit Care Nurs. 2022 Feb 1;68:103123.

19. Virani AK, Puls HT, Mitsos R, Longstaff H, Goldman RD, Lantos JD. Benefits and risks of visitor restrictions for hospitalized children during the COVID pandemic. Pediatrics [Internet]. 2020 Aug 1 [cited 2022 Apr 24];146(2). Available from: /pediatrics/article/146/2/e2020000786/36916/Benefits-and-Risks-of-Visitor-Restrictions-for

20. Iness AN, Abaricia JO, Sawadogo W, Iness CM, Duesberg M, Cyrus J, et al. The effect of hospital visitor policies on patients, their visitors, and healthcare providers during the COVID-19 pandemic: a systematic review. Elsevier Public Health Emergency Collection . 2022 Apr 23;

21. COVID-19 South African Online Portal. COVID-19 Corona Virus South African Resource Portal. 2021.

22. Mbunge E. Effects of COVID-19 in South African health system and society: An explanatory study. Diabetes and Metabolic Syndrome: Clinical Research and Reviews. 2020 Nov 1;14(6):1809–14.

For peer review only

23. Hanewinckel R, Jongman HP, Wallis LA, Mulligan TM. Emergency medicine in Paarl, South Africa: A cross-sectional descriptive study. Int J Emerg Med. 2010;3(3).

24. Mayosi BM, Flisher AJ, Lalloo UG, Sitas F, Tollman SM, Bradshaw D. The burden of non-communicable diseases in South Africa. Lancet. 2009;374(9693):934–47.

25. Western Cape Government. Covid-19 Dashboard | Covid-19 Response [Internet]. 2022 [cited 2022 Apr 26]. Available from: https://coronavirus.westerncape.gov.za/covid-19-dashboard

26. Nyasulu J, Pandya H. The effects of coronavirus disease 2019 pandemic on the South African health system: A call to maintain essential health services. Afr J Prim Health Care Fam Med. 2020;12(1).

27. Mendelson M, Booyens L, Boutall A, Cairncross L, Calligaro G, Dave JA, et al. The mechanics of setting up a COVID-19 response: Experiences of the COVID-19 epidemic from Groote Schuur Hospital, Cape Town, South Africa. South African Medical Journal. 2020;110(10).

28. Theron E, Erasmus HC, Wylie C, Khan W, Geduld H, Stassen W. The lived experiences of emergency care personnel in the Western Cape, South Africa during the COVID-19 pandemic: A longitudinal hermeneutic phenomenological study. African Journal of Emergency Medicine [Internet]. 2022 Dec 1 [cited 2022 Nov 30];12(4):410. Available from: /pmc/articles/PMC9420724/

29. Huddlestone L, Turner J, Eborall H, Hudson N, Davies M, Martin G. Application of normalisation process theory in understanding implementation processes in primary care settings in the UK: A systematic review. Vol. 21, BMC Family Practice. BioMed Central Ltd.; 2020.

30. Braun V, Clarke V. Using thematic analysis in psychology. Qual Res Psychol. 2006;3(2):77–101.

31. Schutt R. Investigating the Social World - The Process and Practice of Research [Internet]. 9th edition. 2018 [cited 2021 May 5]. p. 728. Available from: https://us.sagepub.com/en-us/nam/investigating-the-social-world/book254625

32. Berger R. Now I see it, now I don’t: researcher’s position and reflexivity in qualitative research: http://dx.doi.org/101177/1468794112468475. 2013 Jan 3;15(2):219–34.

33. Williams CMA. The identification of family members’ contribution to patients’ care in the intensive care unit: a naturalistic inquiry. Nurs Crit Care. 2005;10(1):6–14.

For peer review only

34. Companion of choice during labour and childbirth for improved quality of care.
35. Herbst N, Kuntz J. Impact of COVID-19 Visitation Restrictions on ICU Communication, Provider Attitudes and Burnout. Chest. 2021;160(4).
36. Voo TC, Lederman Z, Kaur S. Patient Isolation during Infectious Disease Outbreaks: Arguments for Physical Family Presence. Public Health Ethics. 2020 Jul 1;13(2):133–42.
37. Voo TC, Senguttuvan M, Tam CC. Family Presence for Patients and Separated Relatives During COVID-19: Physical, Virtual, and Surrogate. J Bioeth Inq. 2020;1.
38. Axelsson ÅB, Zettergren M, Axelsson C. Good and bad experiences of family presence during acute care and resuscitation. What makes the difference? Vol. 4, European Journal of Cardiovascular Nursing. Eur J Cardiovasc Nurs; 2005. p. 161–9.
39. Robinson OC. Sampling in Interview-Based Qualitative Research: A Theoretical and Practical Guide. Qual Res Psychol. 2014 Jan;11(1):25–41.

For peer review only

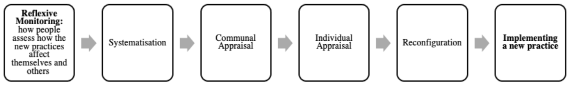

For peer review only

APPENDIX 1: Interview Guide

Thank you for accepting to take part in this interview. Our goal is to learn about your perspective of the changing processes in your trauma care facility during COVID-19, with a particular focus on any changes that have occurred due to no hospital visitors. The interviews will be conducted by Lauren Wiebe, a Master's student from Canada in Public Health Sciences at Karolinska Institutet under the supervision of Dr Helle Alvesson and Dr Willem Stassen.

Experience

A. Prior to COVID-19, what was your role in this unit, and can you describe a bit about your work? For example, what kind of tasks did you do? Who in the unit did you work with? What kind of patients did you see?
B. Before COVID-19, approximately how many patients did you see in a day? How many do you see now? Approximately what month do you feel this changed?
C. Besides COVID-19, during your time working at this hospital have you ever had to work through a secondary emergency before e.g. epidemic, natural disasters, conflict? Have you had this type of experience at another health facility?

Changing practices during COVID-19

A. Can you list some of the practices that have changed in your unit since COVID-19?

→ *The answers from this list can be used as prompts for the below questions. Examples: intake/triage process, referral system, caregiver presence, health seeking behaviour, teamwork among the unit, resource distribution*

B. Please describe what aspects of these changes you value? What aspects do you dislike?
C. Please describe how you learned to adapt to the changing practices? For example, access to training, protocol documents or team meetings.

Making sense of the changes

A. How do these changes compare to your practices before? Can you please provide me with an example?
B. Do you feel like the changes that have occurred are important? If yes, please describe.
C. Do you think your colleagues share an understanding of these changes and their importance? If yes, please describe what influenced this shared understanding? If no, please describe why there are perceived differences?

Participating in the changes

A. In your opinion, do the staff in your unit all equally participate in these changes? If no, what do you think are the main reasons people are not participating in these changes?
B. Are there individuals in the unit who are promoting and supporting the team in these changes? For example, a management team, chief doctor etc.
    a. If yes, please describe the ways they promote/support the unit during COVID?
    b. If yes, did they hold these leadership positions prior to COVID-19?
    c. If there is no central promotor/enforcer, what do you think motivates the staff to participate?
C. Do you feel the new practices are sustainable moving forward? If yes, please explain.

For peer review only

Operationalization of the changes

A. Do you think all staff members in your unit are equally affected by the changes from COVID-19?
B. Since COVID-19, have you seen a change in how the resources are distributed?
C. How have relationships and interactions in your job changed with the changing practices?

Monitoring and assessment of the changes

A. In your opinion have these new practices been effective? Has anything improved from how they were practiced pre-covid? If so, can you give me an example?
B. Please describe how these changes have affected how the medical team works together?
C. How do you feel these changes have affected you individually and your job tasks?
D. Among these changes, what practices do you think could be improved? If so, can you give me an example?

**If not previously discussed-* We would like to ask you a few more questions specifically about the changes you experienced with no caregivers being able to accompany the patient into the health facility.

A. In general, did you notice any changes to care with no caregivers/family being able to accompany the patient?
B. How did the restrictions on family members affect your communication with the patient?
   a. How did you communicate the health news to the families?
   b. How did it influence how you communicated with your colleagues?
C. How did you feel about no caregivers/family members being allowed?
D. What do you think the patients felt having no caregiver with them?
E. In your opinion, what do you feel could have made this better?

Final Questions

A. Are there any other comments you would like to share related to your personal experience working with COVID-19 that have not been addressed in this interview?
B. Do you have any final questions for me?

Questionnaire to be completed before or after the interview:

A. What is your age?
B. What is your identified gender?
C. What is your profession and specialization?
D. How many years have you been working in the health profession?
E. How many years have you been working at this hospital?
F. During the COVID-19 outbreak, were you working part-time or full-time?

For peer review only

APPENDIX 2: Ethical Approval Study II

UNIVERSITY OF CAPE TOWN
Faculty of Health Sciences
Human Research Ethics Committee

Room G50- Old Main Building
Groote Schuur Hospital
Observatory 7925
Telephone [021] 406 6492
Email: hrec-enquiries@uct.ac.za
Website: www.health.uct.ac.za/fhs/research/humanethics/forms

23 February 2021

HREC REF: 052/2021

Dr W Stassen
Division of Emergency Medicine
F51, OMB
Email: willem.stassen@uct.ac.za

Dear Dr Stassen

PROJECT TITLE: THE PERCEPTIONS AND EXPERIENCES OF EMERGENCY MEDICAL STAFF ON THE IMPACT OF COVID-19 ON TRAUMA EMERGENCY CARE SERVICE PROVISION IN THE WESTERN CAPE PROVINCE OF SOUTH AFRICA

Thank you for your response letter, addressing the issues raised by the Faculty of Health Sciences Human Research Ethics Committee (HREC).

It is a pleasure to inform you that the HREC has **formally approved** the above-mentioned study, subject to the following: -

1. There is one outstanding issue. The section on Reflexivity (which seems to be in the wrong place, there is a section on the one visiting master's student. (See below)
   Reflexivity
   Lauren Wiebe has a bachelor's degree in Public Health and Political Science from Vancouver, Canada and is currently a Master's in Public Health Sciences student at Karolinska Institutet. The majority of her experience surrounds public health policy for mental health and infectious diseases for populations in Canada. She has no direct experience working in or with trauma healthcare, reducing preconceptions. However, preconceptions around the role of health workers during the pandemic are that changes have occurred within the trauma unit, potentially in the areas such as teamwork, patient care, protocols and resource distribution.
   Can the researchers please tidy this up and put reflexivity together with the section on credibility and trustworthiness; and put the detail about Lauren in the appropriate place.

This approval is subject to strict adherence to the HREC recommendations regarding research involving human participants during COVID -19, dated 17 March 2020 & 06 July 2020.

Approval is granted for one year until the 28 February 2022.

Please submit a progress form, using the standardised Annual Report Form if the study continues beyond the approval period. Please submit a Standard Closure form if the study is completed within the approval period.
(Forms can be found on our website: www.health.uct.ac.za/fhs/research/humanethics/forms)

Please quote the HREC REF 052/2021 in all your correspondence.

Please note that the ongoing ethical conduct of the study remains the responsibility of the principal investigator.

HREC/REF 052/2021sa

APPENDIX 3: Ethical Approval Dataset I

UNIVERSITY OF CAPE TOWN
Faculty of Health Sciences
Human Research Ethics Committee

Room G50- Old Main Building
Groote Schuur Hospital
Observatory 7925
Telephone [021] 406 6492
Email: hrec-enquiries@uct.ac.za
Website: www.health.uct.ac.za/fhs/research/humanethics/forms

06 August 2020

HREC REF: 386/2020

Dr W Stassen
Division of Emergency Medicine
c/o Ms Vathiswa Mzamo
F-51, OMB
Email: willem.stassen@uct.ac.za

Dear Dr Stassen

PROJECT TITLE: LIVED EXPERIENCES OF EMERGENCY CARE PERSONNEL IN THE WESTERN CAPE, SOUTH AFRICA DURING THE COVID-19 PANDEMIC: A LONGITUDINAL HERMENEUTIC PHENOMENOLOGICAL STUDY

Thank you for submitting your study to the Faculty of Health Sciences Human Research Ethics Committee (HREC) for review.

It is a pleasure to inform you that the HREC has **formally approved** the above-mentioned study.

This approval is subject to strict adherence to the HREC recommendations regarding research involving human participants during COVID -19, dated 17 March 2020 & 06 July 2020.

Approval is granted for one year until the 30 August 2021.

Please submit a progress form, using the standardised Annual Report Form if the study continues beyond the approval period. Please submit a Standard Closure form if the study is completed within the approval period.
(Forms can be found on our website: www.health.uct.ac.za/fhs/research/humanethics/forms)

Please quote the HREC REF in all your correspondence.

Please note that the ongoing ethical conduct of the study remains the responsibility of the principal investigator.

Please note that for all studies approved by the HREC, the principal investigator **must** obtain appropriate institutional approval, where necessary, before the research may occur.

Yours sincerely

PROFESSOR M BLOCKMAN
CHAIRPERSON, FHS HUMAN RESEARCH ETHICS COMMITTEE

HREC 386/2020sa

APPENDIX 4: Consent Letter Study II

PART 1: INFORMED CONSENT FORM

The perceptions and experiences of emergency medical staff on the impact of COVID-19 on trauma emergency care service provision in the Western Cape province of South Africa

Investigators and affiliations
Dr Willem Stassen, Div. Emergency Medicine, University of Cape Town
Prof Helle M. Alvesson, Dept. Global Public Health, Karolinska Institutet
Ms Elzarie Theron, Div. Emergency Medicine, University of Cape Town
Ms Lauren Wiebe, Dept. Global Public Health, Karolinska Institutet

You are invited to participate in a study conducted by the Division of Emergency Medicine at the University of Cape Town and the Department of Global Public Health at the Karolinska Institutet, Sweden.

COVID-19 was declared a global pandemic in March 2020 following a cluster of pneumonia cases in Wuhan, China. Caused by severe acute respiratory syndrome Coronavirus -2 (SARS-CoV-2), COVID-19 continues to spread across the globe. The purpose of this study is to understand the perceptions of emergency care personnel in the Western Cape on the changes in care provision due to COVID-19. We aim to learn from your perspective including, what trauma care processes and practices changed due to COVID-19 and the various ways you had to adjust, specifically inquiring about your perceptions regarding no hospital visitors and escorts. Understanding this perspective can mitigate operational consequences of the COVID-19 pandemic and in the planning for future pandemics. You were selected to participate in this study because you are working clinically on the frontline during the COVID-19 pandemic. Doctors and nurses regardless of baseline qualification are eligible. We aim to recruit a maximum of 20 frontline clinical providers in this study.

Voluntary Participation
Whether you decide to participate in the study or not is entirely your choice and voluntary. There will be no consequences to you if you decide not to participate. You may also decide to change your mind and may withdraw from participating, even if you agreed to it at an earlier stage.

Description of the Process
The interviews will take place over a period of 1-3 months in February-April 2021 and we will require your participation at one session within this period. If you choose to participate in this study, your interview session will take approximately 30-60 minutes. We will require the following:

- A short open-ended survey will be administered and used to collect biographical data at the onset of the study.
- A one-on-one online interview and will take approximately 30 to 60 minutes

For peer review only

Risks or Benefits

Direct benefits to you as an individual could include an opportunity to share your experiences and emotions during the COVID-19 pandemic. It is envisioned that the findings of this study could contribute towards an emergency care system to the advantage of the emergency care community.

Participation in the study could elicit negative emotions due to the sensitive nature of the topic and the emotional experiences you may have with it. In the event that you are experiencing discomfort as a result of any of the research processes, we will support you to obtain the psychological counselling / assistance you require. If you experience psychological discomfort during an interview, please bring this to the interviewer's attention and s/he will support you to obtain the assistance you require.

Confidentiality

All entries will be anonymised during the transcription, analysis and reporting processes. We will use a pseudonym to identify you. The researchers undertake to keep any information provided herein confidential, not to let it out of our possession and to report on the findings from the perspective of the participating group and not from the perspective of an individual.

Right to Refuse or Withdraw

You are not obliged to participate in this study if you do not want to do so. You may withdraw your participation at any time if you choose to do so, up until reporting of analyses. It is entirely your choice and no negative consequences will be incurred if you should choose not to participate or withdraw.

Future research

Anonymised transcribed data will be stored and will later be analysed to inform the initial phase of theory generation, thus contributing towards further related research. Such a theory can assist us in the future to understand the perceptions and experiences of frontline workers and to create systems that may better support them during pandemics.

Who to Contact

The research was reviewed and approved by the Human Research Ethics Committee of the University of Cape Town. The researcher team can be contacted during office hours at elzarie.theron@uct.ac.za. The UCT's Faculty of Health Sciences Human Research Ethics Committee can be contacted on 021 406 6338 in case you have any ethical concerns or questions about your rights or welfare as a participant on this research study.

For peer review only

PART 2: CONSENT

The perceptions and experiences of emergency medical staff on the impact of COVID-19 on trauma emergency care service provision in the Western Cape province of South Africa

I have read the foregoing information, or it has been read to me. I have had the opportunity to ask questions about it and any questions that I have had has been answered to my satisfaction. I consent voluntarily to participate as a participant in this interview. I understand that I may be identifiable.

The purpose of the recordings and the details of its storage and future analysis has been described to me. I have been offered to have any questions that I may have on the recording of the interview – answered and explained. I am giving consent voluntarily and I have been given a copy of this consent form.

Print Name of Participant: ______________________________

Signature of Participant: ______________________________

Date: ______________________________

A copy of this informed consent form has been provided to the participant.

Print Name of Researcher: ______________________________

Signature of Researcher: ______________________________

Date: ______________________________

For peer review only

APPENDIX 5: Consent Letter Study I

Lived experiences of emergency care personnel in the Western Cape, South Africa during the COVID-19 pandemic: a longitudinal hermeneutic phenomenological study

Investigators and affiliations

1. **Willem Stassen**, Division of Emergency Medicine, University of Cape Town
2. **Elzarie Theron**, Division of Emergency Medicine, University of Cape Town
3. **Helena Erasmus**, Department of Psychology, University of South Africa
4. **Craig Wylie**, Division of Emergency Medicine, Stellenbosch University
5. **Waseela Khan**, Division of Emergency Medicine, University of Cape Town
6. **Heike Geduld**, Division of Emergency Medicine, Stellenbosch University

You are invited to participate in a study conducted by the Division of Emergency Medicine at the University of Cape Town.

COVID-19 was declared a global pandemic in March 2020 following a cluster of pneumonia cases in Wuhan, China. Caused by severe acute respiratory syndrome Coronavirus -2 (SARS-CoV-2), COVID-19 continues to spread across the globe. The purpose of this study is to understand the lived experiences of emergency care personnel in the Western Cape. Understanding this perspective is necessary to mitigate adverse psychological and operational consequences of the COVID-19 pandemic by informing strategies to maintain appropriate emergency medical care through the life cycle of this pandemic, and in the planning for future pandemics. You were selected to participate in this study because you are working clinically on the frontline during the COVID-19 pandemic. Doctors, nurses and prehospital staff regardless of baseline qualification are eligible. We aim to recruit 12-16 frontline clinical providers in this study.

Voluntary Participation

Whether you decide to participate in the study or not is entirely your choice and voluntary. There will be no consequences to you if you decide not to participate. You may also decide to change your mind and may withdraw from participating, even if you agreed to it at an earlier stage.

Description of the Process

The study will take place over a period of three months and we will require your participation at various intervals during this period. If you choose to participate in this study, it will take up no more than 60 minutes of your time each week for the duration of the study. We will require the following:

- A short interviewer-administered open-ended survey will be used to collect biographical data at the onset of the study. The survey will be approximately 15 minutes long and will be conducted online, using an online communication tool for video calls.
- You will be asked to share their work schedule during the first interview and follow-up interviews and deadlines for data collection points will be planned accordingly.
- You will be asked to record a 15-30 minute voice recording reflecting on you lived experiences to date in the context of COVID-19. This should be submitted prior to their first pre-shift recording.

For peer review only

- You will be asked to record three to five-minute voice recordings on the following recommended times: 1) prior to a shift, 2) directly after a shift, and 3) 24 to 48 hours after the last shift in a cycle.
- One-on-one interviews will be conducted twice a month for the duration of the study. An online communications platform will be used to conduct the interviews, which will be approximately 40 to 60 minutes in length.
- You will be asked to complete the WHO (Five) Well-being scale every second week. The questionnaire will be available online and consists of a six-point Likert scale measuring positive and negative mental wellbeing.

Risks or Benefits

Direct benefits to you as an individual could include an opportunity to share your experiences and emotions during the COVID-19 pandemic. It is envisioned that the findings of this study will could contribute towards an emergency care system to the advantage of the emergency care community.

You will be reimbursed for the internet data that you use for the study, should you need it.

It is anticipated that the information we gain from this study will help us to reduce adverse psychological and operational consequences of the COVID-19 pandemic by informing strategies to maintain appropriate emergency medical care through the life cycle of this pandemic, and in the planning of future pandemics.

Participation in the study could elicit negative emotions or psychological responses. To monitor this and provide you with the assistance you need in such an event, you will be asked to end your reflections with an indication of how you are currently coping on a scale from 1 to 5 and your need for psychological assistance. In the event that you are experiencing discomfort as a result of any of the research processes, we will support you to obtain the psychological counselling / assistance you require. If you experience psychological discomfort during an interview, please bring this to the interviewer's attention and s/he will support you to obtain the assistance you require.

Confidentiality

In order to ensure that we can link subsequent entries and interviews to previously submitted entries, we need to link your identity. However, all entries will be anonymised during the transcription, analysis and reporting processes. We will use a pseudonym to identify you.

The researchers undertake to keep any information provided herein confidential, not to let it out of our possession and to report on the findings from the perspective of the participating group and not from the perspective of an individual.

Right to Refuse or Withdraw

You are not obliged to participate in this study if you do not want to do so. You may withdraw your participation at any time if you choose to do so, up until reporting of analyses. It is entirely your choice, and no negative consequences will be incurred if you should choose not to participate or withdraw.

Future research

For peer review only - http://bmjopen.bmj.com/site/about/guidelines.xhtml

Anonymised transcribed data will be stored and will later be analysed to inform the initial phase of theory generation, thus contributing towards further related research. Such a theory can assist us in the future to understand the experiences of frontline workers and to create systems that may better support them during pandemics.

Who to Contact

The research was reviewed and approved by the Human Research Ethics Committee of the University of Cape Town. The researcher team can be contacted during office hours at elzarie.theron@uct.ac.za. Should you have any questions regarding the ethical aspects of the study, you can contact the Human Research Ethics Committee of the University of Cape Town, South Africa; contact Tel: +27 21 650 3002, Email: hrec-enquiries@uct.ac.za.

Part 2: Consent

Lived experiences of emergency care personnel in the Western Cape, South Africa during the COVID-19 pandemic: a longitudinal hermeneutic phenomenological study

I have read the foregoing information, or it has been read to me. I have had the opportunity to ask questions about it and any questions that I have had has been answered to my satisfaction. I consent voluntarily to participate as a participant in this interview. I understand that I may be identifiable.

The purpose of the recordings and the details of its storage and future analysis has been described to me. I have been offered to have any questions that I may have on the recording of the interview – answered and explained. I am giving consent voluntarily and I have been given a copy of this consent form.

Print Name of Participant: ____
Signature of Participant: ____
Date: ____

A copy of this informed consent form has been provided to the participant.

Print Name of Researcher: _______________________________________
Signature of Researcher: ____
Date: ____

For peer review only

Standards for Reporting Qualitative Research (SRQR)*

http://www.equator-network.org/reporting-guidelines/srqr/

Title and abstract

**Title** - Concise description of the nature and topic of the study Identifying the study as qualitative or indicating the approach (e.g., ethnography, grounded theory) or data collection methods (e.g., interview, focus group) is recommended	Page 1
**Abstract** - Summary of key elements of the study using the abstract format of the intended publication; typically includes background, purpose, methods, results, and conclusions	Page 2

Introduction

**Problem formulation** - Description and significance of the problem/phenomenon studied; review of relevant theory and empirical work; problem statement	Page 4 and 5
**Purpose or research question** - Purpose of the study and specific objectives or questions	Page 6

Methods

**Qualitative approach and research paradigm** - Qualitative approach (e.g., ethnography, grounded theory, case study, phenomenology, narrative research) and guiding theory if appropriate; identifying the research paradigm (e.g., postpositivist, constructivist/ interpretivist) is also recommended; rationale**	Page 7
**Researcher characteristics and reflexivity** - Researchers' characteristics that may influence the research, including personal attributes, qualifications/experience, relationship with participants, assumptions, and/or presuppositions; potential or actual interaction between researchers' characteristics and the research questions, approach, methods, results, and/or transferability	Page 10
**Context** - Setting/site and salient contextual factors; rationale**	Page 6
**Sampling strategy** - How and why research participants, documents, or events were selected; criteria for deciding when no further sampling was necessary (e.g., sampling saturation); rationale**	Page 7
**Ethical issues pertaining to human subjects** - Documentation of approval by an appropriate ethics review board and participant consent, or explanation for lack thereof; other confidentiality and data security issues	Page 8
**Data collection methods** - Types of data collected; details of data collection procedures including (as appropriate) start and stop dates of data collection and analysis, iterative process, triangulation of sources/methods, and modification of procedures in response to evolving study findings; rationale**	Page 8

For peer review only - http://bmjopen.bmj.com/site/about/guidelines.xhtml

**Data collection instruments and technologies** - Description of instruments (e.g., interview guides, questionnaires) and devices (e.g., audio recorders) used for data collection; if/how the instrument(s) changed over the course of the study	Page 8
**Units of study** - Number and relevant characteristics of participants, documents, or events included in the study; level of participation (could be reported in results)	Page 7 and 8
**Data processing** - Methods for processing data prior to and during analysis, including transcription, data entry, data management and security, verification of data integrity, data coding, and anonymization/de-identification of excerpts	Page 9
**Data analysis** - Process by which inferences, themes, etc., were identified and developed, including the researchers involved in data analysis; usually references a specific paradigm or approach; rationale**	Page 9 and 10
**Techniques to enhance trustworthiness** - Techniques to enhance trustworthiness and credibility of data analysis (e.g., member checking, audit trail, triangulation); rationale**	Page 10

Results/findings

**Synthesis and interpretation** - Main findings (e.g., interpretations, inferences, and themes); might include development of a theory or model, or integration with prior research or theory	Page 10-15
**Links to empirical data** - Evidence (e.g., quotes, field notes, text excerpts, photographs) to substantiate analytic findings	Page 10-15

Discussion

**Integration with prior work, implications, transferability, and contribution(s) to the field -** Short summary of main findings; explanation of how findings and conclusions connect to, support, elaborate on, or challenge conclusions of earlier scholarship; discussion of scope of application/generalizability; identification of unique contribution(s) to scholarship in a discipline or field	Page 15-18
**Limitations** - Trustworthiness and limitations of findings	Page 18-19

Other

**Conflicts of interest** - Potential sources of influence or perceived influence on study conduct and conclusions; how these were managed	Page 20
**Funding** - Sources of funding and other support; role of funders in data collection, interpretation, and reporting	Page 20

*The authors created the SRQR by searching the literature to identify guidelines, reporting standards, and critical appraisal criteria for qualitative research; reviewing the reference lists of retrieved sources; and contacting experts to gain feedback. The SRQR aims to improve the transparency of all aspects of qualitative research by providing clear standards for reporting qualitative research.

For peer review only

For peer review only - http://bmjopen.bmj.com/site/about/guidelines.xhtml

**The rationale should briefly discuss the justification for choosing that theory, approach, method, or technique rather than other options available, the assumptions and limitations implicit in those choices, and how those choices influence study conclusions and transferability. As appropriate, the rationale for several items might be discussed together.

Reference:
O'Brien BC, Harris IB, Beckman TJ, Reed DA, Cook DA. **Standards for reporting qualitative research: a synthesis of recommendations.** *Academic Medicine*, Vol. 89, No. 9 / Sept 2014
DOI: 10.1097/ACM.0000000000000388

For peer review only
